# The carbon footprints of single-use and reusable medical devices: a systematic review

**DOI:** 10.1136/bmjopen-2025-108446

**Published:** 2025-12-19

**Authors:** Amy Booth, Monika Chowaniec, Sanay Goyal, Stuart Faulkner, Sara Shaw

**Affiliations:** 1 Nuffield Department of Primary Care Health Sciences, University of Oxford, Oxford, England, UK

**Keywords:** Climate Change, Systematic Review, Health, Protocols & guidelines

## Abstract

**Abstract:**

**Objective:**

Medical devices account for approximately 6–10% of national health systems’ carbon footprints. The global use of single-use devices has increased, with implications for health systems’ climate impact. This systematic review aimed to synthesise global evidence on medical device carbon footprints, compare single-use and reusable devices and identify lifecycle carbon hotspots to inform policy and practice.

**Design:**

We conducted a systematic review of carbon footprints of medical devices used in clinical settings, reported using Preferred Reporting Items for Systematic Reviews and Meta-Analyses (PRISMA) 2020 guidelines.

**Data sources:**

We searched MEDLINE and Scopus, in 2022 and updated in 2025, and used citation tracking.

**Eligibility criteria:**

English-language, primary research involving carbon modelling of medical devices used in clinical settings was included, with no date restrictions.

**Data extraction and synthesis:**

Articles were screened, and data on carbon modelling methods, device footprints and lifecycle hotspots were extracted by two independent reviewers. Findings were synthesised in figures and tables, and narratively in text. The heterogeneity in carbon modelling approaches prevented quantitative synthesis.

**Results:**

Of 5195 articles identified, 59 met inclusion criteria. Life cycle assessment was the main carbon modelling approach, though application and data quality varied. Carbon footprints of 61 devices were assessed, primarily in surgical (16), anaesthetic (8) and endoscopic (8) specialties. Reusable devices consistently had lower lifecycle footprints. Hotspots were production and manufacturing for single-use devices and reprocessing for reusables.

**Conclusion:**

Reusable devices are preferable from a climate perspective, though efforts are needed to reduce reprocessing emissions. Co-ordinated interventions are required: policymakers can enable supportive regulation; manufacturers can improve device design; healthcare facilities can optimise reprocessing; and providers can prioritise reusable device procurement and use.

**STRENGTHS AND LIMITATIONS OF THIS STUDY**

- This review employed a transparent, replicable search strategy developed with an expert research librarian and reported using Preferred Reporting Items for Systematic Reviews and Meta-Analyses (PRISMA) 2020 guidelines.
- Inclusion criteria were clearly defined, with independent screening by multiple reviewers and conflict resolution by consensus, reducing selection bias.
- Data extraction was conducted independently by two reviewers using a standardised form, increasing consistency and reliability.
- The review was limited to English-language, peer-reviewed primary studies, potentially excluding relevant grey literature or non-English research.
- Quantitative synthesis was not conducted due to substantial heterogeneity in carbon modelling methods, reporting standards and units across studies.

## Introduction

 Anthropogenic climate change, driven by greenhouse gas (GHG) emissions from human activity, poses a major global threat. The health impacts of climate change are well documented, including increases in cardiorespiratory diseases, vector-borne infections and malnutrition.^1 2^ While health systems are strained by climate-related illness, they also contribute to climate change, generating an estimated 5% of global GHG emissions.^3^ Approximately 6–10% of national health system emissions come from medical devices.^3 4^ In 2019, for example, the UK’s National Health Service attributed approximately 2520 kilo-tonnes of carbon dioxide equivalents (CO₂e) to medical devices.^4^

Medical devices are essential to modern healthcare and include instruments, appliances, software or materials used for diagnosis, treatment or care.^5^ In recent decades, health systems globally have become increasingly reliant on single-use devices, intended for one use and then discarded,^6^ rather than reusable devices that can be used across multiple patients and procedures.^7^,^9^ This reliance on single-use devices has implications for the carbon footprint and climate impact of medical devices.

The carbon footprint of medical devices is typically assessed using life cycle assessment (LCA), a methodological approach to evaluate environmental impacts, including GHG emissions, across all stages of a product’s lifecycle, from raw material extraction to disposal.^10^ Within LCAs, two approaches are primarily used for carbon modelling: bottom-up (process-based) and top-down (input–output-based). Bottom-up LCAs rely on detailed, process-specific data and are typically more precise, whereas top-down approaches, such as environmentally extended input–output (EEIO) models, estimate emissions based on financial expenditures, offering less granular insights.^11 12^ Published studies assessing the carbon footprint and hotspots (ie, lifecycle stages that contribute disproportionately to total emissions) of medical devices employ a mix of these approaches, reflecting the growing application of carbon modelling methods in healthcare. Despite a rising number of studies evaluating carbon footprints of medical devices, no comprehensive systematic review has yet synthesised this evidence to inform policy and practice.

Given the urgency to mitigate healthcare’s climate impact, and as literature on medical device carbon footprints expands, there is a need to synthesise this evidence. This paper aimed to (a) systematically review published evidence to summarise medical device carbon modelling approaches (b) report and compare published carbon footprints of single-use and reusable devices and (c) identify carbon hotspots across device lifecycles to inform opportunities for carbon reduction. Our goal was to inform policy and practice by highlighting the climate impact of medical devices, particularly the implications of single-use versus reusable products. Although this review focuses on carbon emissions, we anticipate that findings may have relevance across broader environmental concerns.

Box 1 summarises definitions used in this article.

Box 1 **Climate, carbon modelling and medical device definitions used in article**

**Anthropogenic climate change:** Climate change resulting from human activity, primarily through the emission of greenhouse gases (GHGs) such as carbon dioxide and methane.

**GHG emissions:** Gases that trap heat in the atmosphere, contributing to global warming and climate change. Common GHGs include carbon dioxide (CO₂), methane (CH₄) and nitrous oxide (N₂O). In this review, we use carbon and GHG emissions synonymously.

**Carbon footprint:** The total amount of GHG emissions (expressed as CO₂ equivalents—CO₂e), caused directly and indirectly by an individual, activity, product or organisation.

**Life Cycle Assessment (LCA):** A method used to assess the environmental impact, including carbon footprint, of a product or system over its entire lifecycle, from raw materials to disposal.

**Top-down carbon modelling:** A method (eg, environmentally extended input–output (EEIO)) that estimates emissions based on the monetary cost of a product using sector-level emission factors.

**Bottom-up carbon modelling:** A detailed approach using raw process-specific data across a product’s lifecycle stages to calculate emissions.

**Carbon hotspot:** A stage or component in a product’s life cycle that contributes disproportionately to its total carbon footprint.

**Medical device:** Any instrument, appliance, software, material or other article intended for use in diagnosis, treatment or care.

**Single-use medical device:** A device intended for one-time use, typically made from plastic and discarded after use.

**Reusable/reprocessed device:** A device that undergoes processes after use to ensure its safe reuse across multiple patients or procedures, including cleaning, disinfection, sterilisation and related procedures as well as testing and restoring the technical and functional safety of the used device.

## Methods

We conducted a systematic review of published literature on carbon footprints of medical devices and report the review in accordance with Preferred Reporting Items for Systematic Reviews and Meta-Analyses (PRISMA) 2020 guidelines.

### Eligibility criteria

We included peer-reviewed, English-language, primary research articles that calculated the carbon footprint of medical devices used in clinical settings. All carbon modelling methodological approaches were eligible, and no date restrictions were applied. Studies were excluded if (a) they analysed a medical procedure or health service, rather than a medical device; (b) devices examined were used in public, community and home settings; our focus was on devices used in clinical settings where device procurement, use and reprocessing are centralised and amenable to policy and practice interventions; and (c) the publication was unavailable or not empirical research.

### Information sources and search strategy

We searched MEDLINE and Scopus databases using a strategy developed with a senior research librarian (online supplemental table A.1). Search terms covered two domains: ‘medical devices’ and ‘carbon footprint’”, including MeSH terms where applicable. The search was initially conducted in 2022 and updated in January 2025. Additional sources included citation tracking and a pre-existing database of sustainability-focused healthcare literature. This database was curated by the lead author (AB) to map emerging evidence on environmental impacts of healthcare, including medical devices, as part of broader sustainable healthcare research and was used in this review to identify relevant studies not captured through standard searches.

### Selection process

Titles and abstracts were screened independently by three reviewers (AB, MC, SG). All available full texts were obtained for articles that met inclusion criteria or required further clarification. Articles were managed using EndNote 20, with duplicates removed prior to screening. Disagreements on inclusion were resolved through collective review and discussion with a fourth reviewer (SS).

### Data collection and analysis

Data were independently extracted by AB and MC using a standardised form adapted from similar reviews.^13 14^ Extracted data included: (a) study metadata (title, authors, year, setting); (b) medical device type; (c) carbon modelling approach (eg, boundaries, data sources, assumptions); (d) reported carbon footprint per functional unit (ie, quantified description of the function of the product) of single-use and reusable devices where available; (e) carbon hotspots; and (f) study conclusions. For studies assessing multiple environmental impacts, only carbon data were extracted.

Descriptive data, such as geographical location, device types, modelling approaches and footprint results, were summarised in tables and figures, with narrative synthesis in the text. To provide an overview of carbon modelling approaches, we employed, with permission, a quality assessment tool (online supplemental table A.2) developed by Rizan *et al.*^13^ This tool included criteria such as clarity of functional unit, boundary definitions, data sources, emission factors and transparency of assumptions which we report carbon modelling approaches against.

We report carbon footprint values and units as presented in the original studies, without standardised conversions, to reflect variation in reporting and preserve methodological assumptions. Due to heterogeneity in devices and modelling approaches, no quantitative synthesis was performed. Illustrative examples are included in text, such as comparisons of single-use and reusable device carbon footprints, and studies extrapolating results to national or global levels. Carbon hotspots were reported in studies across lifecycle stages in various formats (eg, without values, as CO₂e, or percentage of contribution). Where available, we recorded the top three hotspots per study, categorised under raw material extraction, production/manufacturing, packaging, transport, use, reprocessing and disposal, to ensure consistency and focus on the most significant reported emission sources.

### Patient and public involvement

No patients or public were involved in this research.

### Ethics statement

Ethical approval was not required for this study as it is a systematic review of previously published literature and does not involve human participants or the collection of new data.

## Results

### Overview of selected studies

**Figure 1:**
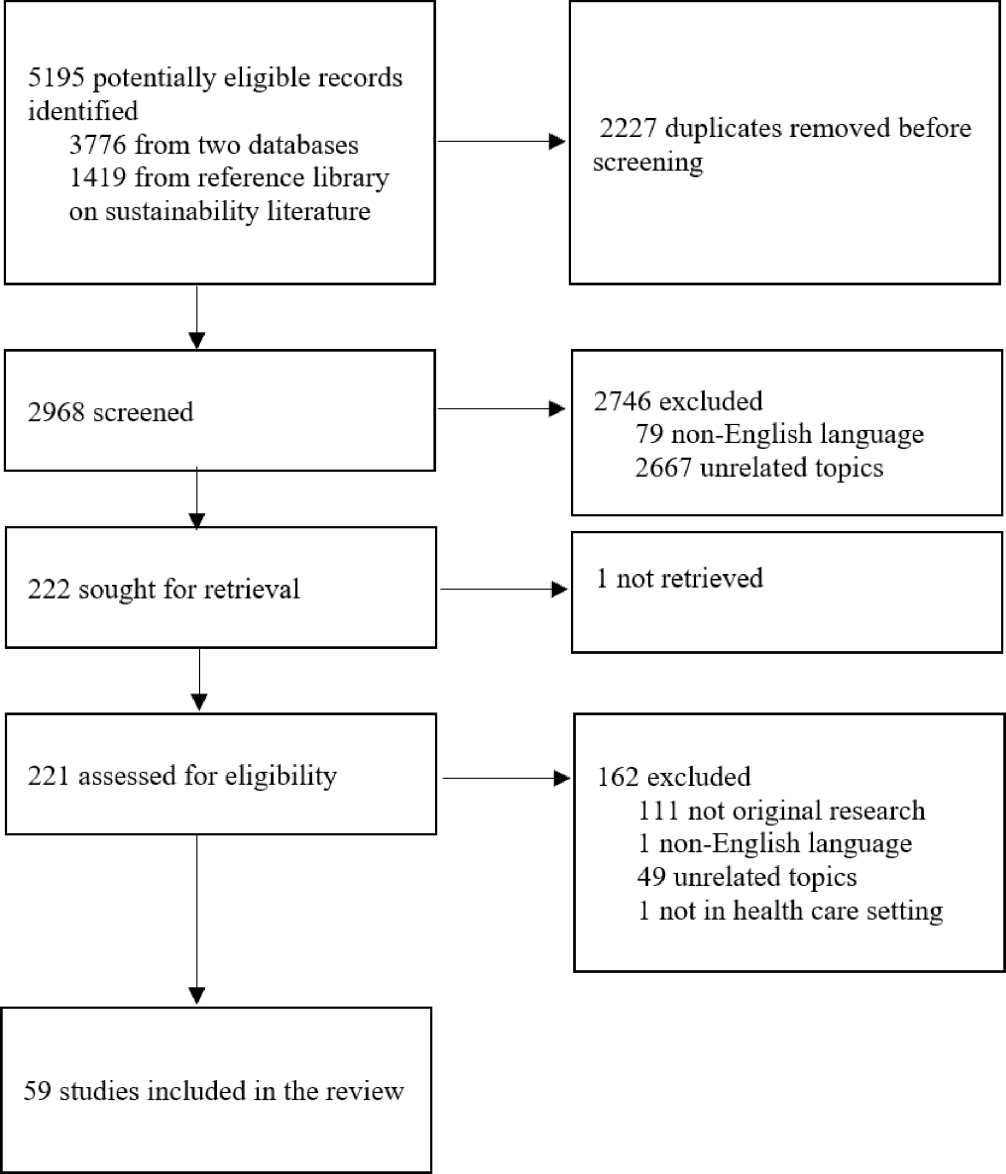
PRIMSA flow diagram of included studies assessing the carbon footprint of medical devices. PRISMA, Preferred Reporting Items for Systematic Reviews and Meta-Analyses,

**Figure 2:**
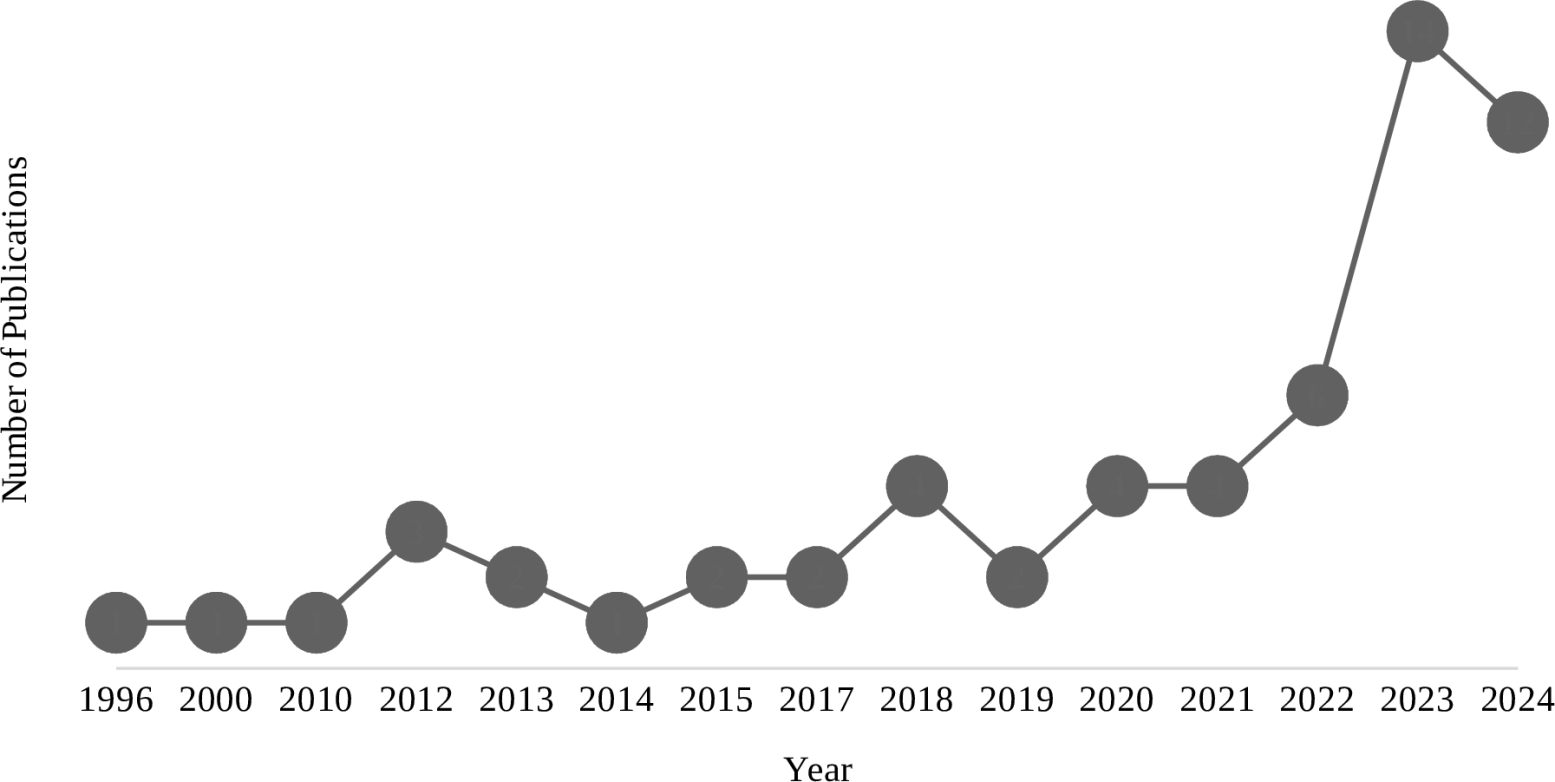
Annual change in number of publications assessing the carbon footprint of medical devices.

A total of 5195 articles were identified, including 3776 from database searches and 1419 from the pre-curated sustainability library (figure 1). After removing 2227 duplicates, 2968 articles remained. Title and abstract screening excluded 2746 articles, 79 due to non-English language and 2667 for irrelevance, leaving 222 for full-text review. One article comparing single-use and reusable drapes was excluded as the full text could not be retrieved despite librarian support and the abstract information was insufficient.^15^ Of the 221 full texts assessed, 162 were excluded for being non-original research (n=111), non-English (n=1), irrelevant (n=49) or outside a clinical setting (n=1). 59 studies met inclusion criteria (see table 1 and online supplemental table A.4 for complete overview of included studies). These were published between 1996 and 2024, with a sharp rise from 2022 (figure 2). Most were conducted in the USA (n=22),^16^,^37^ followed by Australia (n=7),^38^,^44^ the UK (n=6),^45^,^50^ France (n=5)^51^,^55^ and Germany (n=4).^56^,^59^

**Table 1:** Summary of nine studies included in the review (1996–2014), carbon modelling approaches employed and carbon footprint outcomes. Summary of remaining studies can be found in Table A.4

Study, country	Medical device studied (brand)	Carbon modelling approach (guideline)	Functional unit	Carbon footprint		Top three carbon hotspots (% or carbon dioxide equivalents/ CO_2_e if available)		Study conclusions
				Single-use	Reusable	Single-use	Reusable	
Kummerer *et al*,^56^ Germany	Laparotomy pads (not mentioned)	LCA (1980s ‘technical standard’)	One laparotomy pad; results reported for 1000 pads	304.8 kg CO_2_; 225.9 g CO; 1637·9 g N₂O; 565.8 g NOₓ; per 1000 pads	157.1 kg CO₂; 163.9 g CO; 102.9 g N₂O; 432.2 g NO*_x_* per 1000 pads if reusable pads each used 15 times	Production	Washing	Reusable pads have lower carbon footprint
Ison *et al*, ^45^ UK	Suction receptacles (not mentioned)	LCA (not mentioned)	Average kilograms of waste from body fluids produced during 1 year of elective surgery at a district general hospital	Results presented on logarithmic scale	Results presented on logarithmic scale	Not mentioned	Washing	Carbon footprint of single-use suction receptacles greater than reusable device Carbon footprint of reusable affected by washing process
McGain *et al*, ^38^ Australia	Anaesthetic drug trays (Single-use: Chinese-made polyurethane. Reusable: Australian-made reusable nylon tray)	LCA (ISO 14040)	One anaesthetic tray	126 g CO_2_ per tray 204 g CO_2_ per tray with cotton/paper	110 g CO_2_ per tray	Polyurethane tray (111 g CO_2_) Cotton gauze (68 g CO_2_) Polyurethane wrap (8 g CO_2_)	Washing (99 g CO_2_) Drying (9 g CO_2_) Nylon tray (2 g CO_2_)	Significant carbon savings by converting to reusable trays Carbon savings exceed 50% in hospitals using gas co-generation compared with brown coal Adding more cotton and paper to trays increases their carbon emissions
Eckelman *et al*,^16^ USA	LMA (Single-use: Unique, Hangzhou China. Reusable: Classic, Singapore)	LCA (ISO 14040)	Maintenance of airway by 40 disposable LMAs or 40 uses of 1 reusable LMA	0.3 kg CO_2_e per maintenance of one airway	0.2 kg CO2e per maintenance of one airway	Production and polymerisation of PVC	Natural gas production and combustion to produce steam for autoclave machine	Reusable LMAs have lower carbon footprint Carbon footprint of reusable LMA can be reduced by bulk autoclaving, energy-efficient machines and ship transport
Grimmond *et al*,^17^ USA	Sharps containers (Single-use: BD Franklin Lakes. Reusable: Daniels Sharpsmarts Inc)	LCA (British Standards Institute)	Conversion of single-use sharps containers to reusable for 500 uses over 12-month period, workload normalised per 100 occupied bed years	24.2 Mt CO₂e per 100 occupied bed years	4 Mt CO_2_e per 100 occupied bed years	Manufacture (56.4%) Transport (35.8%) Disposal (7.8%)	Decanting and washing (52.5%) Transport (25.5%) Manufacture (15.4%)	Reusable sharps containers reduced GWP by 83.5% over the study period Electricity source can alter global warming potential by 15% Total savings of 64 000 Mt CO_2_e if results expanded across the USA
McGain *et al*,^39^ Australia	CVC insertion kits (not mentioned)	LCA (ISO 14040)	One CVC kit	407 g CO_2_e per kit (European energy)	1211 g CO_2_e per kit (brown coal) 436 g CO_2_e per kit (hospital cogeneration) 764 g CO_2_e per kit (US energy) 572 g CO_2_e per kit (European energy)	Manufacturing of plastics (70%) Steel (25%)	Sterilisation (70%)	Reusable kit has a higher carbon footprint than single-use The energy mix determines the carbon footprint
Ibbotson *et al*,^57^ Germany	Surgical scissors (not mentioned)	LCA (ISO 14040)	4500 use cycles of scissors during 18 years based on technical lifetime of the reusable product	Individual carbon footprints not reported	Individual carbon footprints not reported	Material Manufacturing	Raw material Manufacturing Use	Reusable scissors had the lowest carbon footprint; 11 and 52 times less than plastic and single-use stainless-steel scissors, respectively
Sorensen *et al*,^70^ Denmark	Bedpans (Single-use: Saniwaste System moulded cardboard; GoLoo polyethylene; stainless steel; Supercore T-499 McAirlaid superabsorbent inlay. Reusable: polyethylene)	Consequential LCA (Danish Environmental Design of Industrial Products)	Use of one bedpan once for urinating and defecating while being hospitalised and in bed	0.15–0.2 kg CO₂e per use (cardboard) 0.1–0.15 kg CO₂e per use (polyethylene)	0.25–0.3 kg CO₂e per use (stainless steel) 0.25–0.3 kg CO₂e per use (polyethylene bedpan)	Waste incineration Washing for the cardboard bedpan Incineration for polyethylene bedpan	Washing and disinfection of bedpan. Gloves and disposable bag used to carry bedpans to washroom	Single-use polyethylene bedpan had lowest carbon footprint due to energy recovery opportunity Energy, wastewater management and changed workflow contribute to differences in carbon footprint
Pourzahedi *et al*,^18^ USA	Nanosilver-coated bandage (Acticoat 7)	LCA (not mentioned)	Not mentioned	130.04–130.06 kg CO₂e per bandage	Not mentioned	Silver nanoparticle production (130 kg CO_2_e) Bandage manufacture (0.04–0.05 kg CO_2_e) Bandage disposal (0–0.01 kg CO_2_e)	Not mentioned	Silver nanoparticle synthesis had highest carbon footprint, compared with production and incineration of the bandage

CO₂, carbon dioxide; CO₂e, carbon dioxide equivalent; CVC, central venous catheter; GWP, global warming potential; ISO, International Organization for Standardization; LCA, Life Cycle Assessment; LMA, laryngeal mask airway; Mt, Metric tonne; N₂O, nitrous oxide; NOx, nitrogen oxides; PVC, polyvinyl chloride.

**Figure 3:**
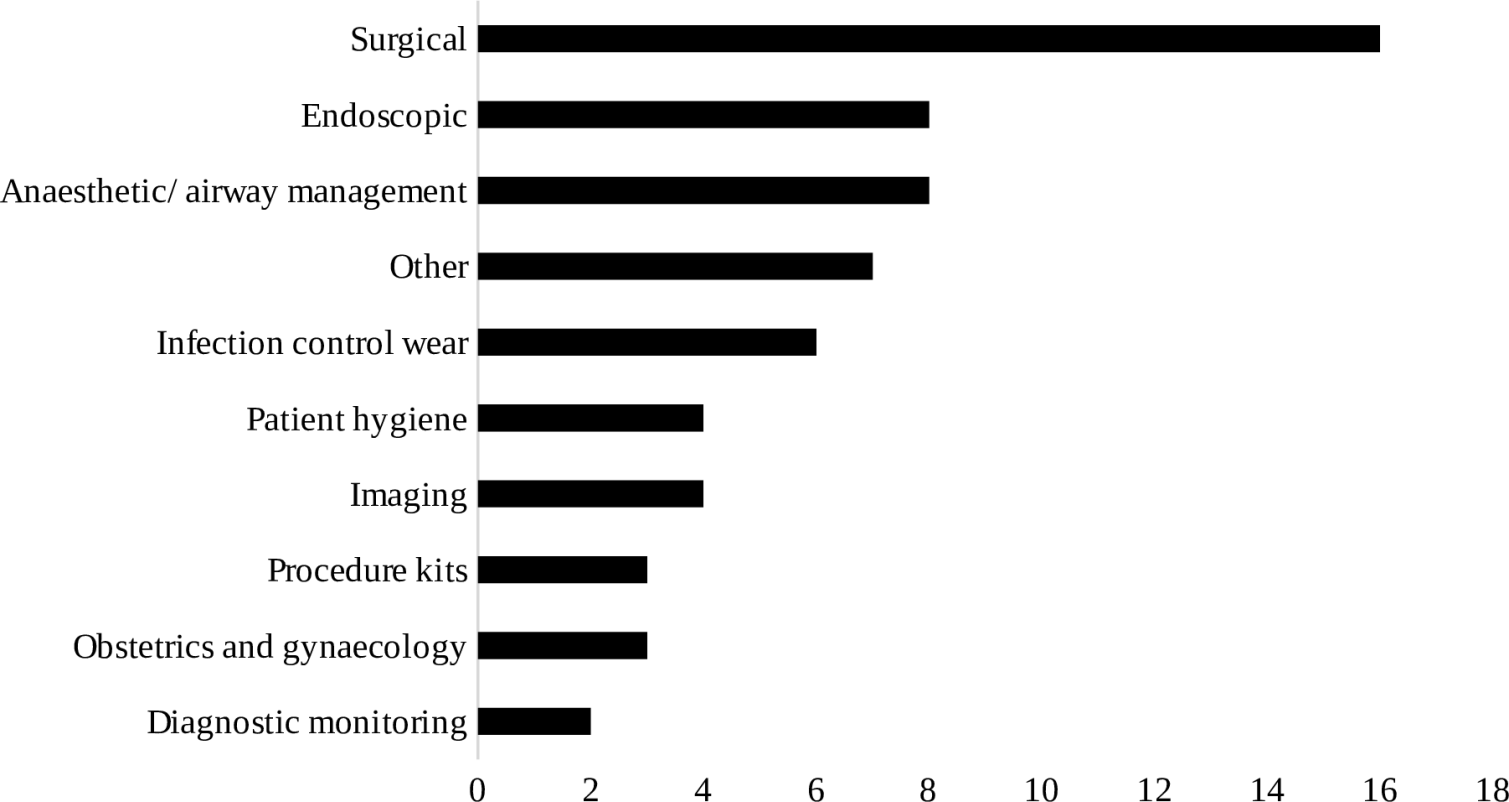
Categories of medical devices with published carbon footprints (1996–2024).

Carbon footprints of 61 distinct medical devices were assessed (figure 3). These included surgical devices (n=16)^2728 30 34 36 45 48 50 51 56^,^58 60^ such as laparoscopic instruments and scrub caps; anaesthetic and airway management devices (n=8)^16 21 35 38 40 42 43 52^ including laryngoscopes; endoscopy devices (n=8)^29 32 41 53 54 59 64 65^ such as ureteroscopes and cystoscopes; infection control wear (n=6)^2247 66^,^69^ including face masks; imaging devices (n=4)^23 37^ including CT and ultrasound scans; patient hygiene products (n=4)^31 33 70 71^ such as bedpans; procedure kits (n=3)^40 49 72^ including central venous catheter insertion kits; obstetric and gynaecological devices (n=3)^19 20 26^ such as vaginal specula; diagnostic monitoring devices (n=2)^25 73^ including blood pressure cuffs; and other devices (n=7)^17 18 24 44 46 74 75^ such as intermittent compression sleeves.

### Carbon modelling approaches

57 studies employed LCA approaches (table 1 and online supplemental table A.4), including consequential LCAs (modelling environmental impacts from change in practice), attributional LCAs (measuring direct environmental burdens) and process-based LCAs. The remaining two studies employed bottom-up carbon footprinting^44^ and product material analysis,^62^ respectively. A complicated landscape of LCA guidelines was evident across studies (box 2). 12 studies reported using ISO 14040,^1619^,^21 25 38^ 5 cited ISO 14044^34 48 50 61 74^ and 11 reported both standards.^22 31 33 42 51 53 59 63 66 68 69^ Others included ISO 14025,^60^ ISO 9001/14001^54^ and ISO 14067.^67^ Additional frameworks included BSI,^17^ PAS 2050,^46^ PEF,^47^ IMPACT 2002+,^72^ Danish EDIP^70^ and GREET.^36^ Three studies mentioned using ‘standardised guidelines’ without naming them,^41 56 73^ and one used a literature-derived protocol.^71^ 16 did not specify the guideline used.^1823 24 26^,^30 32 35 37 43 45 49 52 64^

Box 2 **Overview of Life Cycle Assessment (LCA) frameworks used in studies**

**ISO 14040 (1997, revised 2006)**

Outlines the general principles and framework for conducting LCA.

**ISO 14044 (2006)**

Provides specific requirements, guidelines and methodologies for conducting LCAs, typically used alongside ISO 14040.

**ISO 14025 (2006)**

Standard for communicating quantified environmental information (eg, through Environmental Product Declarations or EPDs) based on LCA.

**ISO 14067 (first published 2013, updated 2018)**

Specifies principles, requirements and guidelines to quantify and report the carbon footprint of products.

**ISO 14001 (first published 1996, updated 2004, latest version 2015)**

Provides a framework for organisations to manage their environmental responsibilities through an Environmental Management System.

**ISO 9001 (first published 1987, updated 2008, latest version 2015)**

Globally recognised standard for quality management. It is sometimes referenced in studies related to environmental processes due to its emphasis on process control and continual improvement.

**British Standards Institute (BSI) Guidelines** UK national standards offering guidance on sustainable practices and environmental management.

**Greenhouse Gas Protocol Publicly Available Specification (PAS) 2050** Global specification for assessing life cycle greenhouse gas emissions of goods and services, widely used in carbon footprinting.

**Product Environmental Footprint (PEF) Methodology** An EU-developed approach to measure the environmental performance of products across their lifecycle.

**Impact assessment of chemical and particulate-related chemical toxicity (IMPACT) 2002+** Life cycle impact assessment method evaluating multiple environmental impacts of chemicals, including global warming potential.

**Environmental Design of Industrial Products (EDIP)** A Danish-developed methodology (1997) for assessing the environmental impacts of products across their life cycle.

**Greenhouse gases, Regulated Emissions, and Energy use in Technologies (GREET) Model** Developed by Argonne National Laboratory, US Department of Energy, estimates greenhouse gas and pollutant emissions related to transportation fuels and vehicle technologies.

SimaPro (n=31)^1618 21 23 25 26 29 30 33^,^35 38^ was the most commonly used LCA software (online supplemental table A.3), followed by OpenLCA (n=5)^36 47 65 71 75^ and Umberto (n=2).^58 74^ Other software included GaBi,^68^ WinPepi,^24^ the Packaging Industry Research Association’s Environmental Management System,^45^ a custom-built Excel-based model^46^ and an online carbon calculator.^28^ Sixteen studies did not report which software was used.^17 19 20 22 27 31 32 41 44 49 56 59 62 64 67 76^ Inventory data sources (eg, for emission factors) varied, with many studies using EcoInvent (n=38)^1618^,^21 25 26 29 30 33^; national or regional databases (n=20)^17^,^1921 23^ including the US Life Cycle Inventory, the UK’s Department for Environment, Food and Rural Affairs, the EU’s International Life Cycle Data system and China’s Life Cycle Database; and industry datasets (n=10).^17 19 20 24 38 39 48 50 59 66^ Eight studies did not specify their inventory source.^28 32 36 41 45 49 52 76^ Data collected for carbon modelling varied and included manufacturer-supplied information (eg, product material composition), manual measurements (eg, energy use, device weight), literature and device usage surveys.

Studies reported various carbon modelling system boundaries (online supplemental table A.3). Most studies reported including raw material extraction (n=45)^1216 18^,^22 24^; production and manufacturing (n=53)^16^,^3538^; packaging (n=17)^16 18 21 22 24 29 30 32 35 38 39 58 61 68 71 73 77^; transport (n=45)^16^,^2426 27 29^; use, referring specifically to the carbon footprint associated with the operation or clinical application of the device (n=37)^1216 18^,^20 23 25^; reprocessing of reusable devices, referring to the cleaning, disinfection and sterilisation of devices for safe reuse, typically on-site or by third-party contractors (n=38)^1617 19 21 22 24^,^27 29^; and disposal and end-of-life management (n=54).^116^,^22 24^

Common assumptions (online supplemental table A.3) included lifespan and reprocessing factors for reusable devices (eg, machine electricity source, machine load and water use per wash), transport mode for device distribution (eg, by air, sea or road) and clinical equivalency between single-use and reusable devices. Exclusions included infrastructure, machinery maintenance, minor components (eg, ink on packaging), staff transport and auxiliary materials (eg, gloves used during reprocessing). Reported limitations included database constraints, assumptions about user behaviour, narrow study scope and limited generalisability due to varying national electricity grids, procurement and waste management practices.

### Carbon footprints of single-use and reusable medical devices

The carbon footprints of medical devices varied widely (table 1 and online supplemental table A.4) and were reported across diverse functional units such as per use, procedure, patient, provider, year or aggregated values. All studies expressed footprints in CO₂e, though units ranged from grams to tonnes. Single-use devices analysed per use ranged from 5·904 g CO₂e for staple firings for one surgical procedure^62^ to 130·06 kg CO₂e for use of one nano-silver-coated bandage.^18^ Reusable devices analysed per use ranged from 3 g CO₂e per use of a reusable microfibre cloth^75^ to 19·7 kg CO₂e for one MRI scan.^23^

Of the included studies, 47 directly compared single-use and reusable medical devices.^1617 21 22 24^,^27 29^ Of these, 39 (83%)^1617 21 22 24^,^27 29^ found reusable devices had a lower carbon footprint, often dependent on reuse thresholds (ie, reusables became more carbon-efficient after a certain number of uses). Studies reported various carbon savings from switching to reusable devices. For example, Grimmond *et al*^17^ found an 83·5% annual carbon reduction with reusable sharps containers; Vozzola *et al*^27^ reported a 66% reduction using reusable surgical gowns; and Burguburu *et al*^51^ found a 31% drop with reusable scrub suits. Eight studies^17 28 29 48 50 61 63 69^ extrapolated carbon savings to national or global scales. For example, Boberg *et al*^61^ projected a 360 tonne CO₂e saving if half of Germany’s laparoscopic cholecystectomies used reusable trocars; Rizan *et al*^48^ calculated a 396 tonne annual saving in England from hybrid laparoscopic equipment; and Cohen *et al*^63^ estimated global use of reusable head covers could reduce 10 000 tonnes.

Six studies^40 43 53 58 67 70^ found reusables had a higher footprint due to factors like raw material impacts (eg, cotton production for masks), fossil fuel–intensive energy grids driving up reprocessing emissions, carbon-intensive waste management strategies and operational efficiency from single-use devices. For example, Leiden *et al*^58^ showed single-use surgical equipment resulted in shorter operating times that lowered theatre energy use. Davis *et al*^41^ found comparable emissions for single-use and reusable duodenoscopes,^41^ while McGain *et al*^39^ reported varying results depending on the national energy grid.

### Carbon hotspots of single-use and reusable medical devices

**Figure 4:**
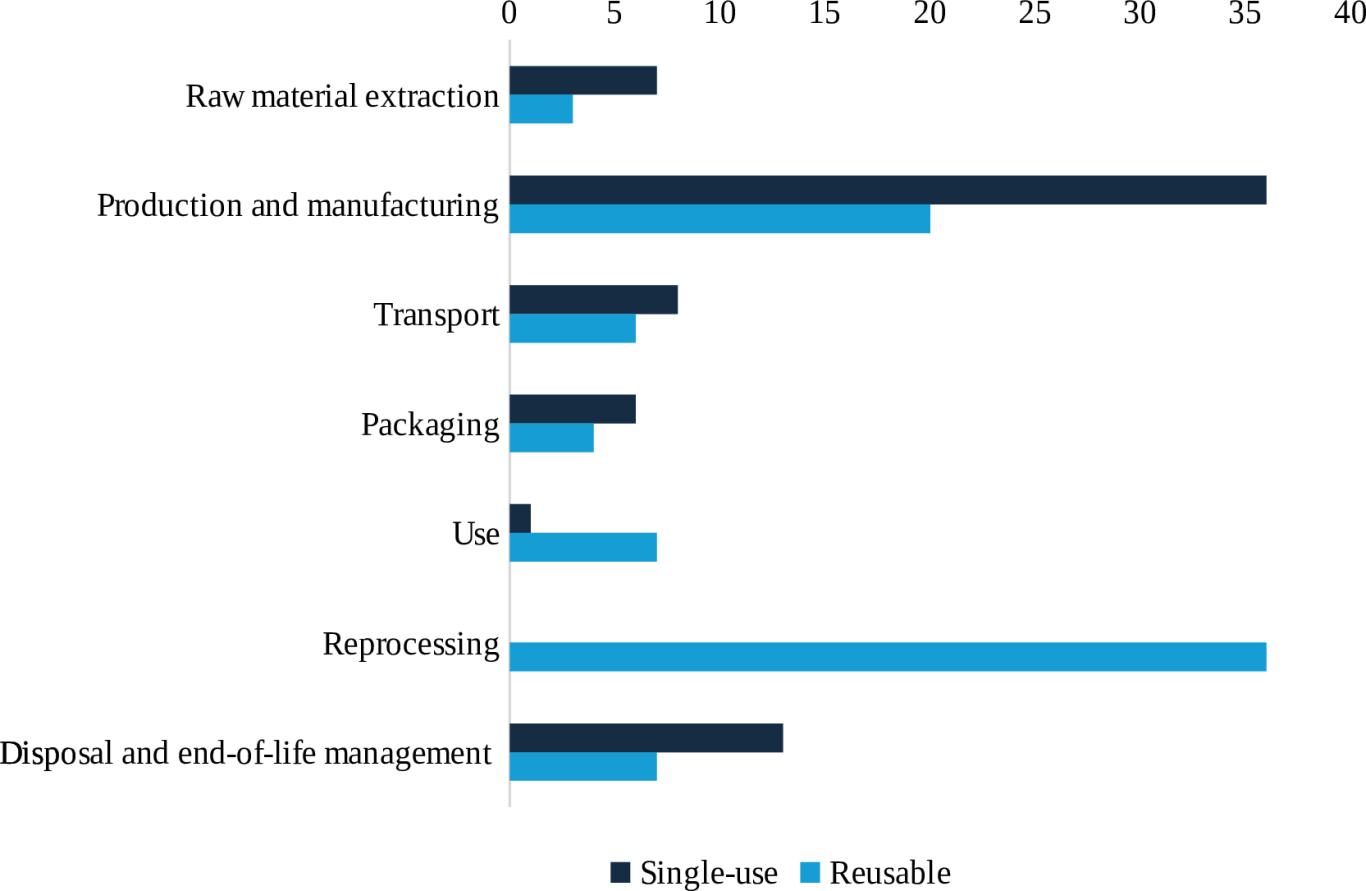
Frequency of lifecycle carbon hotspot mentions for single-use and reusable devices.

Carbon hotspots varied by device type (figure 4). For single-use devices, production and manufacturing were the most frequently cited contributors, identified among the top three in 36 studies.^16^,^1821 22 24^ Examples include manufacture of plastic and steel components in catheter kits (70% and 25%, respectively),^39^ single-use duodenoscopes (91–96%)^29^ and optical fibres in laryngoscopes (64%).^52^ Disposal and end-of-life processes were top hotspots in 13 studies,^17 18 22 24 27 29 33 41 46 48 52 59 70^ contributing, for instance, 14% of the footprint for cholecystectomy instruments.^48^ Transport was a major contributor in nine studies,^24 25 33 46 48 53 60 69 75^ while packaging and raw materials were hotspots in ^22 27 61 68 75^five and ^25 33 47 48 60 67 68^seven studies, respectively, with raw materials, for example, accounting for 40·5% of emissions from face masks.^68^ High production emissions were typically driven by frequent use of devices, while disposal emissions varied with waste treatment method (eg, incineration vs landfill).

For reusable devices, reprocessing, including washing, sterilisation, disinfection and laundry, was the most frequently reported carbon hotspot, appearing among the top three contributors in 36 studies.^1617 21 22 24 26 27 29 31^,^33 38^ For example, reprocessing reusable devices for reuse during the use phase contributed 52.5% of the carbon footprint of reusable sharps containers,^17^ 99% for pulse oximeters^73^ and over 95% for reusable bronchoscopes.^64^ Reprocessing emissions varied with method (eg, autoclaving vs chemical disinfection), reprocessing batch size and national energy mix powering machines (eg, washing machines). Production and manufacturing were top contributors in 18 studies,^1722^,^27 32 41 46 51 53 57 59 61 65 69 75^ for example, representing 74% of the carbon footprint for flexible cystoscopes.^53^ Use-phase emissions were highlighted in five studies,^23 26 29 51 57^ for example, from electricity use of CT scans.^23^ Disposal and end-of-life processes were cited as key contributors in five studies,^33 46 53 69 75^ such as 12% of emissions from flexible cystoscopes.^53^ Transport appeared among the top three hotspots in five studies,^17 24 26 46 53^ particularly when off-site reprocessing was required. Packaging was cited in five studies^21 27 32 52 61^; while raw material extraction was a major contributor in two studies,^33 57^ such as cotton for reusable operating room bed covers.^33^

## Discussion

Our systematic review provides a timely synthesis of evidence on carbon footprints of medical devices, offering three key contributions with implications for policy and practice. First, we make a methodological contribution by mapping the diverse carbon modelling approaches used across studies. While LCA was the predominant approach, its application varied widely in terms of system boundaries, data sources, assumptions and reporting units. This lack of standardisation limits comparability and highlights the need for more consistent yet adaptable methodologies that reflect the diversity of devices and settings. Second, we confirm that reusable devices generally have lower carbon footprints, especially when used extensively throughout their lifespan and reprocessed efficiently (eg, using energy-efficient machinery). However, this was not universally true. In some settings, reusables had higher footprints, highlighting the importance of device- and context-specific assessments rather than blanket assumptions. Third, by identifying recurring carbon hotspots, such as production and manufacturing for single-use devices and reprocessing for reusables, we pinpoint where carbon reduction efforts can be most effectively directed.

Application of LCAs varied considerably across studies included in our review. Particular challenges arose in modelling hybrid devices, such as endoscopes with both reusable and disposable components, and in accounting for diverse reprocessing pathways, including on-site versus third-party sterilisation. Further limitations stemmed from the lack of detailed emissions data for specific geographies, niche device components and sterilisation materials. Restricted access to proprietary data from medical device manufacturers also constrained bottom-up modelling approaches, leading many studies to rely on less accurate proxy data. Efforts are underway, nationally and internationally, to standardise carbon modelling in healthcare and expand emissions databases.^79^ These initiatives have the potential to enhance the accuracy, comparability and utility of carbon footprint data. However, given the diversity of device types and clinical settings, any standardised approach must retain flexibility and be sensitive to local conditions. Importantly, growing academic interest in device carbon modelling should not delay action. While robust data is useful, focusing excessively on measurement risks slowing the implementation of carbon reduction strategies.

Our review demonstrated a convincing climate case for prioritising reusable medical devices. Across diverse global contexts, reusable devices consistently exhibited lower carbon footprints. Alongside this, reusable devices offer additional environmental benefits, including waste reduction and long-term cost savings.^25 38 39^ In the few instances where single-use devices had lower emissions, this was attributable to contextual factors, such as energy-intensive local reprocessing systems or fossil fuel-dominated national electricity grids. In addition to these structural challenges, however, cultural and regulatory barriers constrain widespread adoption of reusable devices. Healthcare provider concerns about infection control, driven by industry marketing, ingrained habits and risk aversion, continue to limit uptake.^80^ These concerns persist despite a lack of strong evidence that single-use devices offer superior infection control.^77 81^ Regulatory complexity presents another obstacle. In some countries, for example, China, reprocessing remains prohibited.^82^ Encouraging, globally regulatory frameworks are evolving. Countries, including the USA, Canada, Japan and Australia, now permit the reprocessing of single-use devices.^83^ The European Union’s (EU’s) Medical Device Regulation similarly allows reprocessing under defined conditions, although national implementation may vary.^84^ For example, at the time of authorship, France is in consultation to determine if they will allow reprocessing; meanwhile, the Netherlands and Germany permit reprocessing, though the Netherlands imposes a more stringent administrative burden. Such divergence constrains economies of scale and limits the circularity potential of medical device reprocessing across EU markets. Meanwhile, in the UK, the Medicines and Healthcare products Regulatory Agency recognises re-manufacturing as a regulated process to restore devices for reuse.^6 84 85^

Beyond prioritising reusable devices, targeting carbon hotspots across device lifecycles offers further opportunities to reduce device emissions, requiring coordinated stakeholder action. Since production and manufacturing often drive emissions, there are opportunities for manufacturers to reduce footprints by optimising design and manufacturing processes, guided by circular economy principles.^6^ Facilities can invest in energy-efficient reprocessing and streamline reprocessing protocols (eg, bulk autoclaving). Healthcare providers can be trained to use devices efficiently, for example, by reducing overuse in procedural packs.^86^ Reducing disposal emissions requires collaboration with waste management systems to improve recycling and reprocessing. Supportive policy at national and local levels is needed to enable and incentivise use of reusable devices. Given the global nature of medical device supply chains, coordinated policy efforts could have a transformative impact on device sustainability.

This review supports prioritising reusable medical devices and encourages transitioning without waiting for complete carbon data on every product. A key exception is low-income and middle-income countries, where device carbon modelling is lacking. Given differing infrastructure and energy profiles, further context-specific research is needed to guide mitigation strategies. Future research should also focus on optimising lifecycle stages, such as improving reprocessing and disposal, and identifying policy, regulatory, economic or behavioural levers to support adoption. For example, although more than half of the studies incorporated reprocessing within their carbon modelling boundaries, these typically referred to on-site or small-scale processes rather than large-scale commercial reprocessing. As such, regional differences in reprocessing regulatory environments, market dynamics and large-scale reprocessing practices were not captured in this review and warrant future investigation. Gaps also remain in understanding industry-level mitigation efforts and infection control outcomes of single-use versus reusable devices. As carbon modelling becomes more common in healthcare, robust quality appraisal standards will be essential for interpreting emerging evidence. Finally, while our review primarily assessed the carbon footprint associated with medical devices, there is a need for future work to integrate a ‘handprint’ approach, that is, examining the procedural performance of the device, and how device use influences patient outcomes and may reduce the need for further, more carbon-intensive care if the device were not used. Such analysis would capture more holistic climate and clinical co-benefits and trade-offs. For example, the findings reported by Leiden *et al*,^58^ where single-use surgical equipment shortened operating times and reduced theatre energy use, illustrate how procedural efficiency can generate positive handprint effects that offset some or all of the carbon footprint of devices.

Our review has several limitations. Literature is concentrated in surgical and anaesthetic specialties and largely driven by a small group of researchers, limiting generalisability across device types. Most studies were conducted in high-income countries; differences in healthcare infrastructure, clinical practice and energy systems mean footprints in low-income and middle-income countries may differ. Although most studies used LCAs, methodological rigour varied, limiting comparability and preventing quantitative synthesis. No validated quality appraisal tool currently exists for healthcare carbon footprint studies; we adapted an existing framework, but its applicability to complex or mixed-use devices was limited. Lastly, our review focused on carbon emissions, limiting assessment of environmental trade-offs and co-benefits between single-use and reusable devices.

## Conclusion

Our review demonstrates that carbon modelling of medical devices is increasingly represented in the academic literature; however, carbon modelling approaches are marked by methodological and data inconsistencies. Despite these inconsistencies, our review confirmed that reusable medical devices tend to have a lower carbon footprint than their single-use counterparts. We further identified carbon hotspots, which differ between device types. Production and manufacturing dominate emissions for single-use devices, while reprocessing contributes the most to emissions from reusable devices. The findings from this review support the preferential adoption of reusable devices and call for policy and practice interventions across the medical device lifecycle to reduce their climate impact. To achieve this, engagement is required by multiple stakeholders. Manufacturers must design and supply reusable products, healthcare facilities need to invest in and optimise reprocessing systems, procurers must prioritise sustainable purchasing, while healthcare providers should be supported to use reusable devices. Regulatory and policy frameworks that facilitate the safe reuse and reprocessing of medical devices are critical to supporting these practices. Addressing this global challenge also requires international cooperation across medical device supply chains. Future research should shift from generating additional carbon footprint data to exploring how existing evidence can inform policy, procurement and clinical practice to reduce the climate impact of medical devices.

## Supplementary material

10.1136/bmjopen-2025-108446 online supplemental file 1

## Data Availability

All data relevant to the study are included in the article or uploaded as supplementary information.
