# Supplementary material for: The carbon footprints of single-use and reusable medical devices: a systematic review
Source: BMJ Open. 2025 Dec 19;15(12):e108446. doi: 10.1136/bmjopen-2025-108446 (PMC12716512; doi:10.1136/bmjopen-2025-108446)
Supplement: online supplemental file 1 [file bmjopen-15-12-s001.docx]

**The carbon footprints of single-use and reusable medical devices: A systematic review**

**Supplementary material**

**Table A.1: Search strategy for studies on carbon footprints of medical devices 2**

**Table A.2: Adapted assessment of carbon modelling approaches 3**

**Table A.3: Carbon modelling approaches used to evaluate medical devices4**

**Table A.4: Summary of all studies included in the review, carbon modelling approaches employed, and carbon footprint outcomes…………………………………………………………………………………..….13**

**Table A.1: Search strategy for studies on carbon footprints of medical devices**

|  | Search terms | Number of articles |
| --- | --- | --- |
| 1 | equipment and supplies/ or air filters/ or exp bandages/ or capillary tubing/ or exp catheters/ or exp contraceptive devices/ or exp culture media/ or exp diagnostic equipment/ or disposable equipment/ or durable medical equipment/ or exp "equipment and supplies, hospital"/ or exp feminine hygiene products/ or gamma cameras/ or gas scavengers/ or gastric balloon/ or humidifiers/ or exp incubators/ or exp infant equipment/ or exp infusion pumps/ or intermittent pneumatic compression devices/ or microbubbles/ or exp "nebulizers and vaporizers"/ or exp needles/ or exp optical devices/ or exp oxygenators/ or exp "prostheses and implants"/ or exp protective devices/ or exp "radiation equipment and supplies"/ or exp radio frequency identification device/ or exp reagent kits, diagnostic/ or exp self-help devices/ or exp sensory aids/ or exp surgical equipment/ or exp surgically-created structures/ or exp syringes/ or exp thermometers/ or exp tomography scanners, x-ray computed/ or exp tourniquets/ or exp transdermal patch/ or exp ventilators, mechanical/ or exp x-ray film/ or exp x-ray intensifying screens/ | 1429511 |
| 2 | anesthesiology/ or exp pathology/ or exp specialties, surgical/ | 307627 |
| 3 | Equipment Reuse/ | 3286 |
| 4 | ((reusable or re-usable or durable or multiuse or multi-use) and (disposable? or single-use)).ti. | 405 |
| 5 | ((reusable or re-usable or durable or multiuse or multi-use) adj5 (disposable? or single-use)).ti,ab,kf. | 912 |
| 6 | ((medical or clinical or health* or diagnos* or surg* or anaethe* or anesthe* or radiolog* or imaging or laboratory or patholog*) adj3 (device? or equipment or supplies or technolog* or instrument* or item? or kit? or clothing)).ti,ab,kf. | 207839 |
| 7 | ((steril* or reusable or re-usable or durable or multiuse or multi-use or disposable or single-use or hybrid) adj3 (device? or equipment or supplies or consumable? or technolog* or instrument* or item? or kit? or clothing)).ti,ab,kf. | 11880 |
| 8 | ((laboratory or patholog* or radiolog* or imaging or surg* or anaesth* or anesthe*) adj2 (department? or dept? or service? or unit?)).ti,ab,kf. | 65740 |
| 9 | 1 or 2 or 3 or 4 or 5 or 6 or 7 or 8 | 1953834 |
| 10 | exp "Conservation of Natural Resources"/ | 123741 |
| 11 | exp climate change/ or greenhouse effect/ | 41623 |
| 12 | carbon dioxide/ or greenhouse gases/ | 104768 |
| 13 | emission?.ti. | 73676 |
| 14 | ((carbon or co2 or gas* or environment* or climat*) adj3 emission?).ti,ab,kf. | 33139 |
| 15 | (emission? adj3 (reduc* or decreas* or chang*)).ti,ab,kf. | 24585 |
| 16 | ((environment* or climat*) adj3 impact?).ti,ab,kf. | 51548 |
| 17 | (greenhouse gas* or green hous* or ghg?).ti,ab,kf. | 19678 |
| 18 | (climat* change or global warming).ti,ab,kf. | 86691 |
| 19 | or/10-18 | 430496 |
| 20 | life cycle.ti,ab,kf. | 52348 |
| 21 | input output analysis.ti,ab,kf. | 436 |
| 22 | cradle to grave.ti,ab,kf. | 350 |
| 23 | ("time in motion" or "time and motion").ti,ab,kf. | 2659 |
| 24 | 20 or 21 or 22 or 23 | 55555 |
| 25 | 19 and 24 | 7185 |
| 26 | Carbon Footprint/ | 1470 |
| 27 | carbon footprint.ti,ab,kf. | 3371 |
| 28 | ((carbon or co2) adj3 model*).ti,ab,kf. | 4417 |
| 29 | ((carbon or co2) adj3 projection?).ti,ab,kf. | 131 |
| 30 | ((carbon or emission?) adj2 (factor? or content)).ti,ab,kf. | 11125 |
| 31 | ((capital or operational) adj2 (carbon or co2)).ti,ab,kf. | 76 |
| 32 | ((environment* or climat* or ecolog*) adj3 (footprint? or impact? or benefit? or cost? or burden)).ti,ab,kf. | 73392 |
| 33 | or/25-32 | 93147 |
| 34 | 9 and 33 | 2239 |

**Table A.2: Adapted assessment of carbon modelling approaches**

| Category | Scoring System |
| --- | --- |
| Consistency | Is the carbon modelling performed consistent with a recognised carbon footprint guideline?  *Guideline stated, referenced, and used consistently (2); Guideline stated, but not used consistently (1); No guideline stated (0)* |
| Transparency | Are the study objectives and purpose clearly stated?  *Clearly stated (2); Ambiguously stated (1); Not stated (0)* |
|  | Does the study clearly state the GHG emissions determined?  *Included GHG emissions clearly stated (2); Included GHG emissions can be deduced (1); GHG emissions not stated or deducible (0)* |
|  | Does the study indicate where they obtained inventory data from?  *All sources of inventory data clearly stated and referenced (2); Some sources of inventory data stated (1); No indication of source of inventory data (0)* |
|  | Are study assumptions and exclusions clearly stated?  *Assumptions and exclusions are clearly stated (2); Assumptions and exclusions are commented on, but ambiguous (1); Assumptions and exclusions not stated (0)* |
|  | Does the study clearly outline the data points collected per process for carbon modelling of the medical device?  *Data points are clear for all processes (2); Data points are limited, and ambiguous (1); Data points not stated (0)* |
|  | How thorough are the reported GHG results?  *Clearly reported for all processes (2); reported as a single aggregate (1); not reported (0)* |
| Completeness | Does the article indicate the boundaries of analysis for the given medical device?  *Yes, includes all reasonable boundaries (2); Yes, includes limited/ ambiguous boundaries (1); No, does not indicate boundaries (0)* |
| Accuracy | Does the study include primary (bottom-up, raw data) or secondary (top-down, financial data) data?  *Primary data only (2); Primary and secondary data (1); Secondary data only (0)* |

**Table A.3: Carbon modelling approaches used to evaluate medical devices**

| Study (year), country | Software (inventory source), impact assessment database | Boundaries | Data collected | Stated assumptions | Stated exclusions | Stated limitations |
| --- | --- | --- | --- | --- | --- | --- |
| Kummerer et al^1^ (1996), Germany | Not provided (Local database for energy mix in West Germany), Not provided | Raw material extraction; Production/manufacture; Use; Disposal/EOL; Transport | Manufacturing data obtained from manufacturers  Energy, raw material, manufacturing, washing, sterilisation, reprocessing, transport, disposal data collected | Reusable pads used 16 times | Minor chemicals | Limited to energy use; data quality |
| Ison et al^2^ (2000), UK | Packaging Industry Research Association Environmental Management System Software Version 4 (Not provided), Not provided | Raw material extraction; Production/manufacture; Distribution; Use; Disposal/EOL | Weights of product components obtained from manufacturers  Packaging weight obtained  Transport distribution data from manufacturers  Product use data (reason, size, location, contents, washing practices, water/detergent use, product lifetime) from two hospitals  Disposal data from waste contractors and water company | Not stated | Transport, capital equipment | Not stated |
| McGain et al^3^ (2010), Australia | SimaPro (EcoInvent; Industry; Local data where available), Not provided | Raw material extraction; Production/manufacture; Packaging; Transport; Use; Disposal/EOL | Weighed trays manually  Water and electricity usage by washer measured | 300 uses lifespan; Miele washer holds 80 trays | None listed | Used European data for China |
| Eckelman et al^4^ (2012), USA | SimaPro 7.3.2 (EcoInvent v2.2), Building for Environmental and Economic Sustainability v4.02 | Raw material extraction; Energy resources; Production/ manufacture; Packaging; Transport; Use; Reprocessing; Disposal/EOL | Materials identified from manufacturing information and weighed  Disposal via municipal waste bins  Reusable cleaning process via high-level disinfection observed | Assumed manufacturing process  Overseas transport via ship; truck on land  No difference in efficacy | Ink on packaging | Proxies from literature |
| Grimmond et al^5^ (2012), USA | Not mentioned (American Chemistry Council Data; Plastics Europe; US industry data; California and Michigan eGrid values; GaBi), Not provided | Production/ manufacture; Transport; Reprocessing; Disposal/EOL | Single-use made in California with wind energy, reusable made in Michigan with coal energy  Biohazardous single-use containers autoclaved and landfilled; chemotherapy containers incinerated  Reusable containers robotically opened, cleaned, and landfilled  Primary energy data for reusable manufacture, washing, and single-use autoclaving, transport  Container size, brand, and quantity data obtained from hospital | Reusable used 500 times | Infrastructure/assets | Not stated |
| McGain et al^6^ (2012), Australia | SimaPro (Local/ international databases), not provided | Raw material extraction; Production/manufacture; Energy sources; Packaging; Transport; Reprocessing; Disposal/EOL | Weighed items manually  Material composition from manufacturer and confirmed with burn test  Volume of water and electricity used by washer/ steriliser measured  Waste disposal using industry data | 300-use lifespan; metal replaced every 100 uses | Common consumables excluded | Country-specific data unavailable |
| Ibbotson et al^7^ (2013), Germany | SimaPro (EcoInvent 2.2.; Australian database;  International Energy Agency database), ReCiPe | Raw material extraction; Production/manufacture; Energy sources; Transport; Use; Reprocessing; Repair; Disposal/EOL | Data from European medical company or literature | Repair every 750 cycles; incineration | None listed | Input data quality; energy mix variance |
| Sorensen et al^8^ (2013), Denmark | SimaPro 7.3.2 (EcoInvent; Danish database), Danish EDIP | Production/manufacture; Energy sources; Reprocessing; Disposal/EOL | Weighed bedpans manually  Standard washing and disinfecting practices for bedpans  Weight and energy use of single-use from manufacturer  Data on excreta from literature  Data on Danish wastewater treatment process | Bedpan reprocessing; bedpan lifespan 1000 uses; bedpan used 3x/day; extreta contents; wastewater treatment | Transport of single-use bedpan | Not stated |
| Pourzahedi et al^9^ (2014), USA | SimaPro 8.1. (US EI database; EcoInvent), TRACI 2.1. | Raw material extraction; Production/manufacture; Transport; Packaging; Use; Disposal/EOL | Production data traced back to raw material extraction  Bandage production modelled on commercially available dressings  Textile fabrication, packaging, transport included  EOL assessed: incineration or sterilisation  Incineration of silver nanoparticles modelled | 100% silver conversion; 90% gas recycling | Excluded supplies (e.g., gloves) | Not stated |
| Campion et al^10^ (2015), USA | Not provided (EcoInvent; USLCI database;  Industry database), TRACI 2v4.0 | Raw material extraction; Production/manufacture; Transport; Use; Reprocessing; Disposal/EOL | Products in custom packs separated, weighed, categorised  Materials identified through manufacturing data and literature.  Two EOL scenarios assessed: landfill and laundry reuse | Laundry process. | Not stated | Reusable not assessed; data availability; health outcomes of packs |
| Esmaeili et al^11^ (2015), USA | Not provided (Not provided), Not provided | Energy sources; Reprocessing | CT energy use data collected from two hospitals  Power multiplied by CT scan idle/active times  Consumable data collected for 23 patients | Not stated | HVAC | Only two machines assessed |
| McGain et al^12^ (2017), Australia | SimaPro (EcoInvent v2.1), ReCiPe | Raw material extraction; Production/manufacture; Transport; Use; Repair; Disposal/EOL | Equipment data from hospitals  Weighed equipment with electronic balance  Measured washer/steriliser utility usage | Washer loads; all anaesthetics done via general anaesthesia | Not stated | Not stated |
| Unger et al^13^ (2017), USA | Not provided (Literature; EcoInvent 2.2.) TRACI | Raw material extraction; Production/manufacture; Packaging; Use; Disposal/EOL | Waste audit of 62 hysterectomies considered  Biopolymer substitution of weight of plastics modelled | Not stated | Not stated | Not stated |
| Davis et al^14^ (2018), Australia | Not provided (Online databases), Not provided | Production/manufacture; Reprocessing; Repair; Disposal/EOL | Data obtained on manufacturing, sterilisation, repackaging, repair and disposal of single-use and reusable scopes from previously validated models  Typical use of reusable in hospital obtained  Washing information taken as usual practice in hospital | Reusable has 16 uses before repair; 180 before decommissioning | Not stated | Data availability; conducted at one site |
| Martin et al^15^ (2018), USA | SimaPro 8.2.3. (National emission database), TRACI | Production/manufacture; Energy sources; Use | Data from manufacturer for single-use and reusable scopes  Reusable washing information from hospital practices  Energy consumption estimated from user manuals  Lighting/HVAC data from standard practice guides  Volume of helium estimated from product guides | MRI active during idle periods; examination time; equipment life-expectancy; number of individuals present for examinations | Costs, personnel, packaging, shipping, and disposal | Only examined one brand of each machine; energy use locally specific; production phase data |
| Sherman et al^16^ (2018), USA | SimaPro 8.1 (EcoInvent v2.2; US database), TRACI | Raw material extraction; Production/manufacture; Energy sources; Packaging; Transport; Reprocessing; Repair; Disposal/EOL | Weighed blades and handles  Transport distances from distributor data  Disinfection energy, chemical, water use from washer/autoclave specs  EOL modelled based on US data | Batteries replaced every 40 uses for reusable; lifetime of reusable 4000 uses; transport by ship and truck; equivalent efficacy of devices | Not stated | Not stated |
| Vozzola et al^17^ (2018), USA | Not provided (Environmental Clarity Inv. LCI database), Not provided | Raw material extraction; Production/manufacture; Packaging; Transport; Reprocessing; Repair; Disposal/EOL | Material composition and weights from manufacturer and gowns sampled to confirm manufacturer specifications  Measured sample of packaging  Laundry process based on field data  Landfill based on literature | Reusable gowns used 60 times before disposal; laundry process and energy use | Wood pallets for shipping | Not stated |
| McPherson et al^18^ (2019), USA | WinPepi v11.65 (American Chemistry Council Data; Industry data; eGrid; US database), Not provided | Production/manufacture; Energy sources; Packaging; Transport; Reprocessing; Disposal/EOL | Data collected on resin manufacture, energy inputs, washing, autoclaving, transport, water, wash products, cardboard, and incineration  Ancillary reusables (e.g. pallets) calculated per trip by lifespan  Container data from hospitals; polymer weight measured  Transport fuel assessed from well to wheel | Reusable used for 500 uses (41.7 years); lifespan of containers; location of polymer manufacture; EOL scenario | Capital machinery, infrastructure, vehicle life-cycle, labour, sharps container contents, non-GHG emissions | Assumptions on location of manufacturer; emission factor database |
| Willskytt et al^19^ (2019), Sweden | OpenLCA (EcoInvent 3.3.; Swedish EOL treatment data; Swedish energy data), ReCiPe | Raw material extraction; Production/manufacture; Energy sources; Packaging; Transport; Use; Reprocessing; Disposal/EOL | Site-specific data used for product assembly and production  Material production and washing data from literature  Manufactured in Sweden, fixed in Denmark  Waste managed using generic Swedish end-of-life data | Pants washed 20 times before disposal; packaging boxes were recycled; washing process | Recycling of used materials | Not stated |
| Donahue et al^20^ (2020), USA | SimaPro 8.5.2. (EcoInvent 2.2; IDEMAT; GREET; EPA WARM), Not provided | Raw material extraction; Production/manufacture ; Energy sources; Transport; Use; Reprocessing; Disposal/EOL | Materials from manufacturer data; weights measured manually  Transport data from manufacturer and industry sources  Disinfection and sterilisation followed manufacturer guidelines | Production process; location of raw materials; transport via ship or truck; equal efficacy | High-level disinfectants, inks, bulk packaging, lubrication, autoclave production | Production assumptions; life cycle data |
| Leiden et al^21^ (2020), Germany | Umberto NXT (Ecoinvent 3.1.), ReCiPe | Raw material extraction; Production/manufacture; Packaging; Transport; Use; Reprocessing; Disposal/EOL | Manufacturing based on weight, material, and design Reusable made in Indiana, disposable in Switzerland Both shipped to Germany and distributed to hospitals Transport distance estimated via Google Maps Washing and sterilisation data collected manually Both types incinerated after end of life | 300 surgeries; 10% instruments loss annually | None listed | Not stated |
| Sanchez et al^22^ (2020), USA | SimaPro 8.1. (EcoInvent v2.2.; US Life Cycle Inventory), TRACI 2.1. | Production/manufacture; Use; Reprocessing; Disposal/EOL | Materials from specs and testing Cuffs disassembled and weighed Packaging weight split per cuff Transport modelled by truck Reusable cuffs assigned ¼ wipe Decontaminated with enzyme bath Disposed via landfill/incineration | Transport; disposal process; equal efficacy; life-time of cuff 3 years | Infrastructure and staff commuting | Location-specific findings; data uncertainty; BP cuff material |
| Vozzola et al^23^ (2020), USA | Not provided (Environmental Clarity Inc LCI Database), Not provided | Raw material extraction; Production/manufacture; Use; Reprocessing; Transport; Disposal/EOL | Market analysis of reusable/disposable gowns Assessed material, packaging, location, laundry, sterilisation, disposal Disposal included collection, transport, processing Reusable: fabric from Europe, assembled in Canada, sent to US Disposable made in China, shipped to US | Gown packaging; laundry process; reusable gowns used 60 times; location of gown production  Assumed three layers of packaging for the gowns. | Not stated | Region-specific database limitations |
| Baxter et al^24^ (2021), USA | EIO-LCA calculator from Carnegie Mellon University (Not provided), Not provided | Raw material extraction; Production/manufacture; Use; Disposal/EOL | Survey of surgeons participating in Wrist and Radius Injury Surgical Trials | Not stated | Not stated | Recall bias; estimated costs of items; EIO-LCA calculator |
| Grimmond et al^25^ (2021), UK | Excel GHG assessment tool (IPCC; GaBi; DEFRA; UK database; US WARM), Not provided | Raw material extraction; Production/manufacture; Energy sources; Transport; Reprocessing; Disposal/EOL | Reusable data from manufacturer: size, model, fill-line, usage Single-use data from baseline trusts Polymer weights from manufacturer Primary data: vehicle size, transport, energy, water, wash inputs Secondary data: polymer production and transport impacts | Location of sourced material; ship and rail transport; material recycled | Capital machinery, infrastructure, vehicle life-cycle, labour, single-use container contents, non-GHG emissions, treatment of container contents, inputs that comprised less than 1% of mass or energy | Region-specific databases; location of production  . |
| Jamal et al^26^ (2021), UK | OpenLCA v1.10.3 (EcoInvent v3.7.1.), Not provided | Raw material extraction; Production/manufacture; Energy sources; Transport; Use; Disposal/EOL | Weighed gloves and packaging materials manually Estimated glove manufacture energy based on machine use Included energy for leak-testing gloves Packaging included Non-sterile gloves manufactured in China, sterile in Malaysia Transport from manufacturer to user, distance estimated via Google Maps EOL: gloves incinerated, packaging recycled | Energy use of glove manufacture; 100 gloves per box; transport via truck and ship; disposal of gloves | Chemicals and oils used to clean and maintain machinery | Assumptions |
| Van Straten et al^27^ (2021), Netherlands | SimaPro 9.1.0.7. (Ecoinvent v3.6), ReCiPe | Production/manufacture; Use; Disposal/EOL | Disassembled one face mask to weigh each component Component info and materials from manufacturer’s data sheet Validation experiment conducted to confirm material composition of filtering fabric | PPE for sterilisation process; 20% rejection rate of gloves; 1000 masks autoclaved per cycle | Production of machinery for manufacturing of face masks and autoclave | Only one type and size of autoclave used; regional database limitations |
| Atilgan Turkmen^28^ (2022), Turkey | GaBi v10.5 (Ecoinvent v3.7.1), CMP 2001 | Raw material extraction; Production/manufacture; Packaging; Transport; Use; Disposal/EOL | Primary data collected from face mask manufacturer in Turkey | Energy mix of Turkey | Not stated | Not stated |
| Boberg et al^29^ (2022), Sweden | SimaPro 9.1.1.1 (EcoInvent v3.6.; Swedish database), Not provided | Raw material extraction; Production/manufacture; Energy sources; Packaging; Transport; Use; Reprocessing; Disposal/EOL | Data on trocar use, hospital processes, and waste management collected via questionnaires Transport distances estimated using Google Maps Road transport modelled as EuroClass 5 lorry Boat transport modelled as freight on ferry Sterilisation machine modelled for water and detergent use EOL modelled as incineration or recycling Included waste scenarios and energy savings from recycling | 500 uses of trocar; trocar autoclave load; EOL process | Machine production. | Only looked at one brand of trocar; uncertainties in modelling and databases. |
| Burguburu et al^30^ (2022), France | SimaPro 8.5 (EcoInvent v3.4), International Life Cycle Data System | Raw material extraction; Production/manufacture; Transport; Energy sources; Use; Disposal/EOL | Reusable scrub suit weighed; materials from manufacturer Single-use suit data from literature Packaging data from manufacturer Reusable suits made in Pakistan and Laos, single-use in China; transported to France Laundry centres located in France | Lifespan of suit assumed to be 63.5 washings | Not stated | Modelling coloration and dying processes; data availability; emission factors for certain components |
| Le et al^31^ (2022), USA | SimaPro 9.1.1.; EPI Suite 4.11; USEtox 2.12  (EcoInvent 3.8.), ReCiPe | Production/manufacture; Energy sources; Packaging; Transport; Use; Reprocessing; Disposal/EOL | Weights obtained from manufacturers Material composition based on similar device (ureteroscope) data Electricity consumption calculated from manual Environmental impact of ICU sepsis patients from reusable scope modelled (literature-based) Energy to reprocess reusable recorded with kilowatt meter Reprocessing machine, detergent, etc., accounted for | Material composition of scopes; reusable used 125 times/year for 5 years; number of ERCPs performed in US annually | Not stated | Model assumptions |
| Maloney et al^32^ (2022), Ireland | OpenLCA (Ecoinvent v.3.7.), Not provided | Production/manufacture; Transport; Use; Reprocessing; Disposal/EOL | Raw materials from manufacturer specifications | Material composition; wipes packaging process; transport via ship then truck; reuse of microfibre 75 times and cotton 60 times; laundry process; disposal process | Machinery upkeep and maintenance, machinery-generated waste | Data availability; assumptions |
| Rizan et al^33^ (2022), UK | SimaPro v9.10. (EcoInvent v3.6; Industry data v.2.0), ReCiPe v1.1 | Raw material extraction; Production/manufacture; Transport; Reprocessing; Disposal/EOL | Raw material composition obtained from manufacturers or expert knowledge Materials weighed Environmental impact of metal and plastic working approximated using global averages Transport determined via supplier discussions EOL via high-temperature incineration | Transport by road; reusable lifespan 500 uses; laundry process; disposal process | Not stated | Assumptions; system boundaries; parameter uncertainty; model uncertainty |
| Agarwal et al^34^ (2023), USA | SimaPro 9.4 (Ecoinvent v.3.8), Not provided | Production/manufacture; Packaging; Transport; Reprocessing; Disposal/EOL | Caps weighed using digital gram scale  Materials identified from manufacturer data | Surgical trainee cap usage; lifespan of cap 300-500 washings | Not stated | Not stated |
| Baboudjian et al^35^ (2023), France | SimaPro v9.3.3. (Ecoinvent v3.5.), Not provided | Raw material extraction; Production/manufacture; Transport; Reprocessing; Disposal/EOL | Scope life cycle inventory from manufacturer  Waste management modelled via French model | Raw material extraction, production and assembly, distribution, maintenance and repair, collection, end-of-life treatment for reusable scopes was considered as having environmental impact close to zero | Not stated | Reusable scope analysis limited to reprocessing; limited generalisability; entire patient pathway not assessed |
| Byrne et al^36^ (2023), UK | Not provided (Not provided), Not provided | Raw material extraction; Production/manufacture; Transport; Reprocessing; Disposal/EOL | Trays measured and weighed individually Procurement amounts of pulp trays for the past three years from GSTT/Trust sourcing team | Quantity of plastic trays to replace the use of pulp trays | Not stated | Not stated |
| Cohen et al^37^ (2023), Netherlands | SimaPro v9.1.0.7 (Ecoinvent v3.6), ReCiPe 2016 v1.1 | Production/manufacture; Use; Transport Reprocessing; Disposal/EOL | Data from suppliers | 100 reuses before disposal | Not stated | Limited generalisability |
| Duffy et al ^38^(2023), Canada | SimaPro v9.2.0.2 (Ecoinvent 3.8), TRACI 2.1. | Raw material extraction; Production/manufacture; Energy sources; Packaging; Transport; Reprocessing; Disposal/EOL | Reusable data scaled per use based on 1-year lifespan, plus 1 cleaning wipe per patient Materials data from manufacturers, deconstructing device to find weight of individual parts 1/4 of cleaning wipe modelled per use | Daily use; equivalent power use between devices | LED sensor and cable, gold plating covering pin header of cable | Overestimation of cleaning phase of reusable devices; data availability |
| Griffing and Overcash^39^ (2023), USA | Not provided (Environmental Genome and Environmental Clarity Inc), TRACI 2.1. | Raw material extraction; Energy sources; Production/manufacture; Transport; Reprocessing; Disposal/EOL | Electricity modules based on 2019 US average grid data Life cycle inventories used to map manufacturing steps Survey of large laundry systems to determine average cycles before removal Patient day and APD data used to produce disposable verse reusable ratio | Lifespan of reusable pads; frequency of pad change | Not stated | Not stated |
| Hemberg et al^40^ (2023), Sweden | SimaPro 9.2.0.2 (Ecoinvent 3.6), IMPACT 2002+ | Raw material extraction; Transport; Disposal/EOL | Weight of all single-use and reusable metal items measured by scale Single-use and reusable items modelled based on supplier data Washing/sterilisation process modelled from nearby laundry facility Energy and water consumption data from autoclave and washer disinfector manufacturer | Transport distances assumed via Google Maps | Not stated | Not stated |
| Kemble et al^41^ (2023), USA | Not provided (Not provided), Not provided | Production/manufacture; Packaging; Transport; Use; Reprocessing; Disposal/EOL; PPE used during reprocessing | Single-use manufacturing data from manufacturers Reusable data from institutional device performance database Transportation costs based on nautical miles and 26.5g CO_2_/ton-km | Case volumes; EOL process; PPE for reprocessing | Detergents for reprocessing | Limited generalisability |
| Lichtnegger^42^ et al (2023), Austria | Umberto (Ecoinvent 3.8), ReCiPe 2008, UBP 2013, CML 2016; Environmental Footprint 3.0 method | Production/manufacture; Transport; Use; Reprocessing; Disposal/EOL | Iterative process with manufacturer to create a realistic life cycle model Default inventories for transportation processes, not primary data | Reprocessing cycles; impact of reprocessing; transport; packaging | Not stated | Not stated |
| Luo et al^43^ (2023), China | Not provided (China Life Cycle Database (CLCD v.0.7); China Products Carbon Footprint Database (2022), Not provided | Raw material extraction; Production/manufacture; Transport; Use; Disposal/EOL | Primary data surveys from three manufacturing enterprises | Data assumptions; washing process | Not stated | Not stated |
| Meissner et al^44^ (2023), Austria | Not provided (Not provided), Not provided | Lithium production/manufacture | Product material analysis: all components and packaging deconstructed and weighed Total material requirement calculated for each component and stapling system Lithium cells separated and analysed via mass spectrometry | Not stated | Reprocessing; recycling of batteries | Did not conduct LCA of all components of scopes |
| Quintana-Gallardo et al^45^ (2023), Spain | SimaPro v.9.0 (Ecoinvent v3.8), Environmental Footprint v3 | Raw material extraction; Transport | Data on all single-use materials used in the hospital collected to determine composition, weight, origin, and other LCA-relevant details | Transport by lorry; distance from local companies; EOL process | Not stated | Model uncertainty |
| Rouviere et al^46^ (2023), France | SimaPro v9.2.0.1 (Not provided), Not provided | Raw material extraction; Production/manufacture; Transport; Use; Disposal/EOL | Material composition of blades from manufacturers' datasheets. Weights obtained using a calibrated scale | Reprocessing process; lifespan of blade 4000 reprocessing cycles | Not stated | Not stated |
| Snigdha et al^47^ (2023), India | SimaPro 9.2. (Literature; Consultation; EcoInvent), Midpoint (H) V1.03; ReCiPe 2016 and CED (LHV) | Raw material extraction; Use; Disposal/EOL | Literature surveys; field visits; Ecoinvent databases; modelling and interviews with medical textiles, healthcare experts, and waste management facilities | Machinery used in production; product materials; transport distances; energy consumption | Not stated | Lack of standard LCA methodology; region-specific database |
| Chang et al^48^ (2024), USA | SimaPro 9.5.0.1 (Ecoinvent 3; US LCI database), TRACI 2.1. | Raw material extraction; Production/manufacture; Use; Reprocessing; Disposal/EOL | Source and raw material specifications from manufacturers.  Laundry data based on average per pound from utility monitoring (Jan-Sept 2023)  Detergent composition from open-source data | Transport by ship and truck; EOL process | Transport to disposal site | Underestimate of laundry cycles |
| Chen et al^49^ (2024), Australia | SimaPro (Ecoinvent v.3.10), IPCC 2021 GWP100 | Raw material extraction; Production/manufacture; Transport; Reprocessing; Disposal/EOL | Product specifications, place of manufacture, and components by weight obtained from manufacturers or suppliers Generic specifications (‘general plastic’, ‘plastic film’) used where specific details were unavailable All products and their packaging were weighed | Energy mix from fossil fuel | Not stated | Manufacturer use data; assumptions rather than direct audit; variations in brands and energy sources |
| Donahue et al^50^ (2024), USA | SimaPro 9.4.0.3 (Ecoinvent 3.9.1), ReCiPe 1.1.; TRACI 2.1. | Raw material extraction; Energy sources; Production/manufacture; Transport; Use; Reprocessing; Disposal/EOL | Single-use caps deconstructed and components weighed Cardboard packaging weighed and included in LCA Reusable cap manufacturing and composition data not available – caps weighed and average taken Water use per cycle of laundering based on manufacturer and global laundry practices data | Location of material source; transport routes; materials; use of cap | Bulk packaging and home laundry machine production | Overestimated carbon assumptions |
| Hansell et al^51^ (2024), Australia | Not provided (DEFRA), Not provided | Production/manufacture | Devices disassembled and individual components weighed | Not stated | Manufacturing, transport, use, and disposal | Resource constraints precluded LCA |
| Kidane et al^52^ (2024), USA | SimaPro v.9.6 (Ecoinvent v.3.10), TRACI 2.1. v 1.08 | Raw material extraction; Production/manufacture; Packaging; Transport; Use; Reprocessing; Disposal/EOL; PPE use during reprocessing | Material composition for each scope provided by the manufacturer, including transport distances and modes Electrical energy consumption for reusable scope from voltmeter observation study Electrical energy for single-use from maximum battery capacity. Reprocessing protocol observed by members of the study | Approximated functional unit; use patterns; equivalent efficacy of devices | Laryngoscope recycling, repairs, facility emissions, labour emissions | Exclusions skewed results in favour of reusable laryngoscopes; only modelled one reprocessing approach |
| Lehane et al^53^ (2024), USA | OpenLCA (Not provided), US Environmental Protection Agency Greenhouse Gas Equivalencies calculator | Raw material extraction; Production/manufacture; Use; Disposal/EOL | Items weighed  Material assessments based on manufacturer statements | Material composition | Not stated | Not stated |
| Lightfoot et al^54^ (2024), Australia | SimaPro 9.4 (AusLCI v2.42; EcoInvent v 3.9.1.), IPCC 2021 GWP100 | Raw material extraction; Production/manufacture; Transport; Use; Disposal/EOL | Weights of trays obtained from manufacturer's specification sheets. Analysis via custom peer-reviewed Excel tool (physical product data - materials, manufacturing processes, EOL destinations) | Not stated | Packaging, domestic delivery of tray to point of use, use of tray | Only assessed one brand; limited generalisability; conflict of interest |
| Lopez-Munoz et al^55^ (2024), Spain | OpenLCA V.2.0.3 (EF Secondary Data Sets V. EF 2.0), EF midpoint calculator | Raw material extraction; Production/manufacture; Transport; Reprocessing; Disposal/EOL | Material composition analysis performed at Centre for Biomaterials and Tissue Engineering using energy dispersive X-ray analysis, field emission scanning electron microscopy, differential scanning calorimetry, thermogravimetric analysis, and Fourier transform infrared spectroscopy | Transport routes | Transport of raw materials and between port and hospital, manufacturing and assembly, | Limited boundaries included; limited emission factors for incineration |
| Massart et al^56^ (2024), France | Not provided (“Base empreinte” of the “Agence de la transition écologique”, France), Not provided | Raw material extraction; Production/manufacture; Transport; Use; Reprocessing; Disposal/EOL | Elements examined to evaluate components of each final product  Waste elimination estimated using currently used disposal sectorisation | Transport by ship and truck; lifespan assumed 8 years; composition of reusable bronchoscope | Sterilisation, energy use from use and disinfection | Data on composition of bronchoscope; infection prevention quality; limited generalisability |
| Pioche et al^57^ (2024), France | SimaPro V.9.3 (Ecoinvent V.3.8), CML-baseline V3.07. | Raw material extraction; Production/manufacture; Transport; Reprocessing; Disposal/EOL | Single-use material composition data supplied by manufacturer  Reusable manufacturer disclosed material composition | Lifespan of gastroscope assumed 1280 procedures over 6 years; 10-year life-expectancy for endoscopy system | Not stated | Quality of endoscopy and clinical impacts not evaluated |
| Rizan^58^ (2024), UK | SimaPro (Ecoinvent v3.6.; Industry data v.2.0), ReCiPe v1.1.; Endpoint Hierarchist | Raw material extraction; Production/manufacture; Transport; Use; Reprocessing; Disposal/EOL | Raw material composition and weight of hybrid ports provided by supplier and verified with samples Single-use port data from Rizan and Bhutta 2022 | Reuse 200 times; disposal/ EOL process | Not stated | Study funded by supplier of hybrid ports |
| Thöne et al^59^ (2024), Germany | Not provided (Ecoinvent 3.8; Industry data; literature), ReCiPe 2016 | Production/manufacture; Transport; Use; Reprocessing; Disposal/EOL | Direct statements from companies/staff Empirical measurements by investigators Data from literature Estimates based on expert opinions | 133 usages per device and maintenance after every 11th use; quantity of devices per washing cycle; energy mix; packaging | External emission parameters needed for ureteroscopy e.g., anaesthetic gases/electricity for AC | Data only on scope, not operating room; data accuracy; limited generalisability; regional databases |

**Table A.4: Summary of all studies included in the review, carbon modelling approaches employed, and carbon footprint outcomes.**

| Study (year), country | Medical device studied (brand) | Carbon modelling approach (guideline) | Functional unit | Carbon footprint | | Top three carbon hotspots (% or carbon dioxide equivalents/ CO_2_e if available) | | Study conclusions |
| --- | --- | --- | --- | --- | --- | --- | --- | --- |
|  |  |  |  | **Single-use** | **Reusable** | **Single-use** | **Reusable** |  |
| Kummerer et al^56^ (1996), Germany | Laparotomy pads  (not mentioned) | LCA (1980’s “technical standard”) | One laparotomy pad; results reported for 1000 pads | 304·8 kg CO_2_; 225·9 g CO; 1637·9 g N₂O; 565·8 g NOₓ; per 1000 pads | 157·1 kg CO₂; 163·9 g CO; 102·9 g N₂O; 432·2 g NOx per 1000 pads if reusable pads each used 15 times | Production | Washing | Reusable pads have lower carbon footprint |
| Ison et al^45^ (2000), UK | Suction receptacles  (not mentioned) | LCA (not mentioned) | Average kilograms of waste from body fluids produced during one year of elective surgery at a district general hospital | Results presented on logarithmic scale | Results presented on logarithmic scale | Not mentioned | Washing | Carbon footprint of single-use suction receptacles greater than reusable device  Carbon footprint of reusable affected by washing process |
| McGain et al^38^ (2010), Australia | Anaesthetic drug trays  (Single-use: Chinese-made polyurethane. Reusable: Australian-made reusable nylon tray) | LCA (ISO 14040) | One anaesthetic tray | 126 g CO_2_ per tray  204 g CO_2_ per tray with cotton/paper | 110 g CO_2_ per tray | Polyurethane tray (111 g CO_2_)  Cotton gauze  (68 g CO_2_)  Polyurethane wrap (8 g CO_2_) | Washing (99 g CO_2_)  Drying (9 g CO_2_)  Nylon tray (2 g CO_2_) | Significant carbon savings by converting to reusable trays.  Carbon savings exceed 50% in hospitals using gas co-generation compared to brown coal  Adding more cotton and paper to trays increases their carbon emissions |
| Eckelman et al^16^ (2012), USA | Laryngeal Mask Airway/ LMA  (Single-use: Unique, Hangzhou China. Reusable: Classic, Singapore) | LCA (ISO 14040) | Maintenance of airway by 40 disposable LMAs or 40 uses of 1 reusable LMA | 0·3 kg CO_2_e per maintenance of one airway | 0·2 kg CO2e per maintenance of one airway | Production and polymerisation of PVC | Natural gas production and combustion to produce steam for autoclave machine | Reusable LMAs have lower carbon footprint  Carbon footprint of reusable LMA can be reduced by bulk autoclaving, energy efficient machines, and ship transport |
| Grimmond et al^17^ (2012), USA | Sharps containers  (Single-use: BD Franklin Lakes. Reusable: Daniels Sharpsmarts Inc.) | LCA (British Standards Institute) | Conversion of single-use sharps containers to reusable for 500 uses over 12-month period, workload normalised per 100 occupied bed years | 24·2 Mt CO₂e per 100 occupied bed years | 4 Mt CO_2_e per 100 occupied bed years | Manufacture (56.4%)  Transport (35.8%)  Disposal (7.8%) | Decanting and washing (52.5%)  Transport (25.5%) Manufacture (15.4%) | Reusable sharps containers reduced global warming potential (GWP) by 83.5% over study period  Electricity source can alter global warming potential by 15%  Total savings of 64000 Mt CO_2_e if results expanded across USA |
| McGain et al^39^ (2012), Australia | Central Venous Catheter/ CVC insertion kits  (Not mentioned) | LCA (ISO 14040) | One CVC kit | 407 g CO_2_e per kit  (European energy) | 1211 g CO_2_e per kit (brown coal)  436 g CO_2_e per kit (hospital cogeneration)  764 g CO_2_e per kit (US energy)  572 g CO_2_e per kit (European energy) | Manufacturing of plastics (70%)  Steel (25%) | Sterilisation (70%) | Reusable kit has higher carbon footprint than single-use  The energy mix determines the carbon footprint |
| Ibbotson et al^57^ (2013), Germany | Surgical scissors  (Not mentioned) | LCA (ISO 14040) | 4500 use cycles of scissors during 18 years based on technical lifetime of the reusable product | Individual carbon footprints not reported | Individual carbon footprints not reported | Material  Manufacturing | Raw material  Manufacturing  Use | Reusable scissors had the lowest carbon footprint; 11 and 52 times less than plastic and single-use stainless-steel scissors respectively |
| Sorensen et al^70^ (2013), Denmark | Bedpans  (Single-use: Saniwaste System moulded cardboard; GoLoo polyethylene; stainless steel; Supercore T-499 McAirlaid superabsorbent inlay. Reusable: polyethylene) | Consequential LCA (Danish Environmental Design of Industrial Products) | Use of one bedpan once for urinating and defecating while being hospitalised and in bed | 0·15–0·2 kg CO₂e per use (cardboard)  0·1–0·15 kg CO₂e per use (polyethylene) | 0·25–0·3 kg CO₂e per use (stainless steel)  0·25–0·3 kg CO₂e per use (polyethylene bedpan) | Waste incineration  Washing for the cardboard bedpan Incineration for polyethylene bedpan | Washing and disinfection of bedpan.  Gloves and disposable bag used to carry bedpans to washroom | Single-use polyethylene bedpan had lowest carbon footprint due to energy recovery opportunity  Energy, wastewater management, and changed workflow contribute to differences in carbon footprint |
| Pourzahedi et al^18^ (2014), USA | Nanosilver coated bandage (Acticoat 7) | LCA (not mentioned) | Not mentioned | 130·04–130·06 kg CO₂e per bandage | Not mentioned | Silver nanoparticle production (130 kg CO_2_e) Bandage manufacture (0·04-0·05 kg CO_2_e)  Bandage disposal (0-0·01 kg CO_2_e) | Not mentioned | Silver nanoparticle synthesis had highest carbon footprint, compared to production and incineration of the bandage |
| Campion et al^78^ (2015), USA | Custom child-delivery packs  (not mentioned) | LCA (ISO 14040) | One single-use custom pack | 0·1–20 kg CO₂e per pack depending on pack contents | Not mentioned | Quantity of cotton per pack | Not mentioned | The quantity of cotton was the determining factor for the highest carbon footprint in various packs  Newly designed pack including content paper list, gown, under buttocks drape, gauze, umbilical cord clamp, bulb syringe, basin, and pack wrapper would reduce GWP by 80% |
| Esmaeili et al^37^ (2015), USA | Computerised Tomography  (Hospital 1: GE Lightspeed 64 slice 2007. Hospital 2: Philips Brilliance 64-slice 2005) | LCA (not mentioned) | A single scan | Not mentioned | 7·3 kg CO₂e per scan (GE Lightspeed 64 Slice)  5·1 kg CO₂e per scan (Philips Brilliance 64 Slice) | Not mentioned | Energy | Carbon footprint dependent on energy efficiency of machine and part of the body scanned |
| McGain et al^40^ (2017), Australia | Anaesthetic equipment – anaesthetic circuits; face masks, laryngeal mask airway; direct laryngoscope blades and handles; video-laryngoscope blades and handles  (Single-use: Supreme. Reusable: Proseal) | Consequential LCA (ISO 14040) | All anaesthetic equipment used in a single year | 5775 kg CO_2_e per year (all equipment single-use)  5095 kg CO_2_e per year (all equipment single-use except for reusable laryngoscope handles and video-laryngoscopes) | 5575 kg CO₂e per year (all equipment reusable)  6556 kg CO₂e per year (all equipment reusable, except for single-use face masks)  6763 kg CO₂e per year (all equipment reusable, except for single-use direct laryngoscope blades) | Face masks  Laryngoscope blades | Washer electricity  H_2_O_2_ steriliser electricity | Carbon footprint of single-use versus reusable depends on energy source  Using single-use and reusable in combination has highest carbon footprint |
| Unger et al^20^ (2017), USA | Products used in four types of hysterectomies  (not mentioned) | LCA (ISO 14040) | All medical devices that contained petroleum-based plastics suitable for biopolymer substitution for each of the four types of hysterectomies | Individual carbon footprints not reported | Individual carbon footprints not reported | Not mentioned | Not mentioned | Petroleum-based medical devices have lower carbon footprint than biopolymers due to high carbon footprint of agricultural activities to manufacture biopolymers |
| Davis et al^41^ (2018), Australia | Flexible ureteroscopes  (Single-use: LithoVue, Boston Scientific. Reusable: Olympus Flexible Video Ureteroscope) | LCA (“Standardised carbon footprint protocol guidelines”) | One endourologic case | 4·43 kg CO₂e per endourologic case | 4·47 kg CO₂e per endourologic case | Manufacturing (3·45kg CO_2_e)  Sterilisation (0·3kg CO_2_e) Disposal (0·3kg CO_2_e) | Washing/ sterilisation (3·94kg CO_2_e)  Repair (0·31kg CO_2_e)  Manufacturing (0·06kg CO_2_e) | Carbon footprint of single-use and reusable ureteroscopes are comparable |
| Martin et al^23^ (2018), USA | Abdominal imaging  (Ultrasound: GE LOGIQ E9. Computerised Topography-CT: GE Discovery HD750. Magnetic Resonance Imaging-MRI: Philips Ingenia 1.5-T) | LCA (not mentioned) | One abdominal imaging examination divided into active and idle periods | Not mentioned | 6·6 kg CO₂e per examination (CT)  19·7 kg CO₂e per examination (MRI)  1·2 kg CO₂e per examination (ultrasound) | Not mentioned | CT  Production (4 kg CO_2_e)  Use (2·7 kg CO_2_e)  MRI  Use (13·7 kg CO_2_e)  Production (6·1 kg CO_2_e)  US  Use (0·5 kg CO_2_e)  Production (0·6 kg CO_2_e) | US had the lowest carbon footprint during both production and use  MRI had the highest carbon footprint  The operating schedule and energy mix influenced the emissions |
| Sherman et al^21^ (2018), USA | Laryngoscopes  (Single-use polycarbonate blade: Heine XP Disposable. Single-use PVC/ polycarbonate handle: Flexicare BritePro. Single-use steel blade and handle: BOMimed Fiber Optic Blade. Reusable laryngoscope: Rusch Snaplight Fiber Optic/ Blade.) | LCA (ISO 14040) | One handle and one blade | 1·41 kg CO_2_e per plastic handle  1·6 kg CO_2_e per metal handle  0·38 kg CO_2_e per plastic blade  0·44 kg CO_2_e per metal blade | 0·06 kg CO2e per handle (high-level disinfection)  0·08 kg CO2e per handle (low-level disinfection)  0·23 kg CO2e per handle (sterilisation)  0·06 kg CO2e per blade (high-level disinfection)  0·08 kg CO2e per blade (low-level disinfection)  0·22 kg CO2e per blade (sterilisation) | Manufacturing  Device assembly | Reprocessing  Repackaging | Reusable laryngoscope handles and blades have lower carbon footprint  Metal single-use handles and blades have higher carbon footprint than plastic single-use versions  Sterilisation has higher carbon footprint than high-level disinfection for reusable laryngoscope |
| Vozzola et al^22^ (2018), USA | Isolation gowns  (24 gowns from 8 different suppliers: High Five; Kimberley-Clark; Medline; Precept; S2S Global; American Dawn; Encompass; Fashion Seal) | LCA (ISO 14040/ ISO 14044) | 1000 isolation gowns | 310 kg CO₂e per 1000 uses  0·31 kg CO₂e per use | 218 kg CO₂e per 1000 uses  0·218 kg CO₂e per use | Manufacture and delivery of gown (300 kg CO_2_e)  Manufacture and delivery of packaging (6·95 kg CO_2_e)  Landfill of gown/packaging (1·99 kg CO_2_e) | Laundry (146 kg CO_2_e)  Manufacture and delivery of gown (68·6 kg CO_2_e) Wastewater treatment to restore water (2·08 kg CO_2_e) | 30% reduction in GWP by using reusable gowns  Carbon footprint of single-use gown depended on weight, and reusable depended on efficiency of laundry process |
| McPherson et al^24^ (2019), USA | Sharps containers  (not mentioned) | LCA (not mentioned) | Supply of sharps containers for disposal of sharps waste for one year | 8·37 Mt CO₂e per 10 000 adjusted patient days  248·6 Mt CO₂e annually | 2·9 Mt CO₂e per 10000 adjusted patient days  86·2 Mt CO₂e annually | Manufacture (148 Mt CO_2_e) Transport (69·8 Mt CO_2_e)  Treatment and dispose (30·2 Mt CO_2_e) | Transport (77·6 Mt CO_2_e)  Manufacture (3·1 Mt CO_2_e)  Wash (4·9 Mt CO_2_e) | Converting from single-use sharps containers to reusable significantly reduces carbon footprint  Electricity source can alter manufacturing carbon footprint by 82% |
| Willskytt et al^71^ (2019), Sweden | Incontinence products  (not mentioned) | LCA (Bockin et al framework and Environmental Protection Strategies guideline) | Hygiene function of one absorbent product with medium absorption capacity of medium size used for three measures (recycling manufacturing waste, changed material composition, reuse of pant part); hygiene function for one day at the studied ward in an elderly home used for fourth measure (effective use through customisation) | Did not report individual carbon footprints | Did not report individual carbon footprints | Material production | Not mentioned | Single-use pants generate higher emissions than reusable  37% reduction in impact by reusing part of the product; 26% reduction by changing material composition; 21% reduction by effective use of product through customisation; 5% reduction by recycling manufacturing waste |
| Donahue et al^26^ (2020), USA | Vaginal specula  (Single-use: Welch Allyn Kleenspec Disposable Vaginal Specula. Reusable: Skylar Merit stainless steel grade 304 Graves and Pederson specula; surgical grade stainless steel grade 316 specula) | LCA (not mentioned) | Completion of 20 examinations by each type of instrument | 0·88 kg CO₂e per examination | 2·48 kg CO₂e per examination (grade 316 stainless steel)  1·69 kg CO₂e per examination (grade 304 stainless steel) | Material production  Manufacturing | Use/ reprocessing  Manufacturing Transport | Reusable specula had favourable carbon profiles across range of scenarios  Emissions from reusable specula can be reduced by 33-36% by sourcing lower carbon electricity to reprocess instruments and by 11-12% by sterilising instead of autoclaving  Using reusable specula instead of single-use would have reduced carbon emissions by 75% in the hospital examined |
| Leiden et al^58^ (2020), Germany | Spinal fusion surgical instruments  (Single-use: Neo Pedicle Screw System, Neo Medical SA.  Reusable: Viper 2 surgical instruments, DePut Synthes) | LCA (ISO 14040) | Specific set of surgical instruments for the realisation of single level lumbar fusion surgery, including the implantation of four screws and two rods by means of a set of surgical instruments | Individual carbon footprints not reported | Individual carbon footprints not reported | Production | Steam sterilisation | Single-use set results in 82% lower carbon footprint compared to reusable, due to ease of use which reduces surgery time and emissions from running the theatre |
| Sanchez et al^25^ (2020), USA | Blood Pressure/ BP Cuff  (Single-use and Reusable: Flexiport, Welch Allyn) | LCA (ISO 14040) | Providing BP readings under 4 different healthcare scenarios: office/clinic; ambulatory; regular ward; ICU | 9·78 kg CO₂e per reading (office/clinic, incineration)  7·20 kg CO₂e per reading (office/clinic, landfill)  4·89 kg CO₂e per reading (ambulatory, incineration)  3·60 kg CO₂e per reading (ambulatory, landfill)  1·96–2·14 kg CO₂e per reading (regular ward, incineration)  1·44–1·58 kg CO₂e per reading (regular ward, landfill)  1·96–2·14 kg CO₂e per reading (ICU, incineration)  1·44–1·58 kg CO₂e per reading (ICU, landfill) | 0·23 kg CO₂e per reading (office/clinic, incineration)  0·18 kg CO₂e per reading (office/clinic, landfill)  0·12–0·34 kg CO₂e per reading (ambulatory, incineration)  0·09–0·27 kg CO₂e per reading (ambulatory, landfill)  0·90 kg CO₂e per reading (regular ward, incineration)  0·71 kg CO₂e per reading (regular ward, landfill)  0·06–0·25 kg CO₂e per reading (ICU, incineration)  0·05–0·20 kg CO₂e per use (ICU, landfill) | Raw material extraction  Manufacturing Transport | Manufacturing of wipes to sterilise | Reusable blood pressure cuffs have lower carbon footprint in all healthcare scenarios |
| Vozzola et al^27^ (2020), USA | Surgical gowns  (not mentioned) | LCA (not mentioned) | 1000 uses of a gown in an operating room setting | 1·636 kg CO₂e per use  1636 kg CO₂e per 1000 gowns | 0·557 kg CO₂e per use  557 kg CO₂e per 1000 uses | Manufacturing (1495 kg CO₂e) Packaging (121 kg CO₂e)  End-of-life (10·9 kg CO₂e) | Laundry (778 kg CO₂e) Manufacturing (143 kg CO₂e) Packaging (76·7 kg CO₂e) | 66% reduction in carbon footprint by using reusable surgical gowns  Energy efficient laundry process has potential to further reduce carbon footprint of reusable gowns |
| Baxter et al^28^ (2021), USA | Single-use surgical supplies used in hand surgery  (not all mentioned) | Economic input-output LCA (not mentioned) | Not mentioned | 7·8–28·8 kg CO₂e per procedure depending on surgeon | Not mentioned | Not mentioned | Not mentioned | Differences in use of drapes, towels and gauze by surgeons contributed to the most variation in carbon outcomes  If all surgeons reduced their supply use, they could decrease the carbon footprint associated with supplies by 800·6 thousand Mt CO_2_ annually |
| Grimmond et al^46^ (2021), UK | Sharps containers  (Reusable: Sharpsmart) | LCA (Publicly Available Specification/PAS 2050) | Total fill line litres of sharps containers needed to dispose of sharps over the respective study years across 40 trusts | 3896·4 Mt CO₂e over 12 months across 40 NHS trusts | 628·9 Mt CO₂e over 12 months across 40 NHS trusts | Manufacture (2179 Mt CO₂e) Treat and dispose (1162·8 Mt CO₂e) Transport (554·2 Mt CO₂e) | Manufacture (116·7 Mt CO₂e) Transport (422 Mt CO₂e) Treat and dispose (31·9 Mt CO₂e) | 83·9% reduction in carbon emissions over the 12-month study period in the 40 hospital trusts by switching to reusable sharps containers |
| Jamal et al^47^ (2021), UK | Sterile and non-sterile medical gloves  (brands anonymised) | LCA (ISO and Product Environmental Footprint/PEF) | An individual clinician using one pair of non-powdered, medium-sized gloves for a healthcare procedure | 6·80e–02 kg CO₂e per pair latex-free non-sterile gloves  7·89e–01 kg CO₂e per pair latex sterile gloves  8·00e–01 kg CO₂e per pair non-latex sterile gloves | Not mentioned | Raw material (non-sterile gloves)  Manufacturing (sterile gloves) | Not mentioned | Non-sterile gloves have the lowest carbon footprint while sterile have the highest  Latex and non-latex sterile gloves have similar impact |
| Van Straten et al^69^ (2021), Netherlands | Face mask  (Aura 1862+ (3M)) | LCA (ISO 14040/ 14044) | Protection of 100 healthcare workers against airborne viruses using one FFP2 certified face mask, each during one working shift of an average of 2 hours in a hospital in the Netherlands | 6·55 kg CO₂e for protection of 100 healthcare workers | 2·77 kg CO₂e for protection of 100 healthcare workers | Production (4·55 kg CO₂e) End-of-Life (0·93 kg CO₂e) Transport (0·65 kg CO₂e) | Production (1·24 kg CO₂e) Sterilisation (0·83 kg CO₂e) End-of-Life (0·35 kg CO₂e) | Lower carbon footprint for reprocessed medical face masks compared to new |
| Atilgan Turkmen^68^ (2022), Turkey | Type I medical face masks  (not mentioned) | LCA (ISO 14040/ 14044) | One 3-layer single-use medical face mask with nose wire | 21·5 g CO₂e per mask | Not mentioned | Raw material (40·5%)  Packaging (30·0%)  Mask production (15·5%) | Not mentioned | Raw material supply is the carbon hotspot for disposable face masks |
| Boberg et al^61^ (2022), Sweden | Laparoscopic trocar  (not mentioned) | Attributional LCA (ISO 14044) | 500 laparoscopic cholecystectomies | 565 kg CO₂e from 500 cholecystectomies | 118 kg CO₂e per 500 cholecystectomies (reusable)  507 kg CO₂e per 500 cholecystectomies (mixed system) | Production and packaging (60-70%) | Production and packaging (60-70%) | Single-use trocar had highest carbon footprint, 379% higher than the reusable system and 12% higher than mixed system  If 50% of the 800000 annual laparoscopic cholecystectomies in Germany are done using reusable systems, 360 Mt CO_2_ could be saved |
| Burguburu et al^51^ (2022), France | Scrub suits  (Reusable: Elis) | LCA (ISO 14040/ ISO 14044) | To provide an operating theatre employee with scrub suits on a daily basis over a 4-year service | 674 kg CO₂e over 4 years of service | 462 kg CO₂e per 4 years of service 31·2 kg CO₂e per scrub suit | Fabric manufacturing (35%) | Fabric manufacturing (48%) Use and reuse (44%) | Reusable scrub suits have 31% lower carbon footprint, due to reduced quantity required  Modification of energy consumption and mix reduces carbon footprint |
| Le et al^29^ (2022), USA | Duodenoscopes  (Single-use: Exalt Model D, Boston Scientific. Reusable with single-use endcaps: TJF-Q190V, Olympus. Conventional reusable: TJF-Q180V, Olympus) | LCA (not mentioned) | One endoscopic retrograde cholangiopancreatography procedure (ERCP) | 36·3–71·5 kg CO₂e per ERCP procedure | 1·53 kg CO₂e per ERCP procedure (reusable)  1·54 kg CO₂e per ERCP procedure (reusable with single-use caps) | Manufacturing (91-96%)  Disposal (3-5%)  Electricity during use | Electricity during use (62%)  Cleaning and disinfection (26%) | Single-use duodenoscope emits 24-47 times more carbon emissions.  If all US facilities used single-use, over 18 million kg CO_2_e would be produced. |
| Maloney et al^75^ (2022), Ireland | Surface wipes and clothes  (not mentioned) | LCA (ISO 14040) | One commercially available cotton, microfibre or single-use polyethylene cloth used by a single clinician to clean down a contaminated surface | 104 g CO₂e per use | 3–7 g CO₂e per use | Transport (42%) Disinfectant (40%) Packaging (4%) | Disinfectant (30% in cotton, 38% in microfibre) Production (24%) Disposal (6%) | Microfibre reusable wipes have a lower carbon footprint than cotton wipes, regardless of disinfectant used, and outperform cotton in microbiological effectiveness |
| Rizan et al^48^ (2022), UK | Hybrid and single-use instruments for laparoscopic cholecystectomy  (Surgical Innovations Ltd.; Microline Surgical Inc.) | Attributional LCA (ISO 14044) | The number of the three types of instruments typically required to perform one laparoscopic cholecystectomy | 2559 g CO₂e per laparoscopic cholecystectomy (clip applier)  1139 g CO₂e per laparoscopic cholecystectomy (scissors)  3495 g CO₂e per laparoscopic cholecystectomy (four ports) | 445 g CO₂e per laparoscopic cholecystectomy (clip applier)  378 g CO₂e per laparoscopic cholecystectomy (scissors)  933 g CO₂e per laparoscopic cholecystectomy (four ports) | Raw material extraction and manufacturing (57%) Transportation (29%) Waste (14%) | Single-use components (62%) Decontamination of reusable parts (37%) | Hybrid versions have 76% lower carbon footprint than single-use instruments, saving 5·4 kg CO_2_e per operation.  If hybrid instruments were used in all laparoscopic cholecystectomies in England, it would save 396 Mt CO_2_e annually  If shipping used instead of air, carbon footprint is reduced by 22-33% for single-use versions |
| Agarwal et al^30^ (2023), USA | Scrub caps  (not mentioned) | LCA (not mentioned) | Providing operating room staff with a scrub cap daily for 1 year | 14 kg CO₂e for one year of scrub caps | 5 kg CO_2_e for one year of scrub caps | Not mentioned | Not mentioned | Carbon footprint of single-use scrub caps was higher than reusable caps  The overall positive carbon footprint of reusable scrub caps depends on reliable and frequent usage of the cap in daily surgical practice |
| Baboudjian et al^53^ (2023), France | Flexible cystoscopes  (Single-use: aS4C, aScope, Ambu. Reusable brand not mentioned) | LCA (ISO 14040/ 14044) | Specific set required for high-level disinfection of cystoscope, including 4 pairs of nitrile gloves, 1 pair of neoprene gloves, apron, wipe, soft brush, sterile swab, 3 sterile sponges, 2 transport bags, camera cover, sterile cover, 3 peracetic acid canisters, 1 sterile water canister | 2·06 kg CO₂e per single-use cystoscope | 3·08 kg CO₂e to reprocess one reusable cystoscope | Production (39%)  Assembly (29%)  Transport (19%) | Production (74%)  Transport (14%)  End-of-life (12%)  (Hotspots of materials used for reprocessing only) | Carbon footprint of full lifespan of single-use cystoscope was 33% lower than carbon footprint of just reprocessing reusable cystoscopes  Sterilisation process offsets the benefit of reusable devices on carbon footprint |
| Byrne et al^49^ (2023), UK | Medical trays  (not mentioned) | LCA (not mentioned) | Trays used in one year | 5292 kg CO₂e for trays used in one year | 5852 kg CO_2_e for trays used in one year | Not mentioned | Not mentioned | During the first year, reusable plastic trays have a higher carbon footprint due to the higher initial manufacturing impact, but emissions lower by the second year  Reusable trays offer long-term carbon savings |
| Cohen et al^63^ (2023), Netherlands | Surgical head covers  (Single-use non-woven vicose: Model Kosak, Mölnlycke. Single-use non-woven polypropylene: Model Annie Green, Mölnlycke. Reusable polyester: Model Selma, CleanLease B.V.) | LCA (ISO 14040/ 14044) | Annual supply of head covers | 1·9 Mt CO₂e for one year of viscose head covers  1·7 Mt CO₂e for one year of polypropylene head covers | 0·7 Mt CO₂e | Manufacturing | Washing | Reusable head covers have 56-61% lower carbon footprint than single-use head covers.  Global use of reusable head covers could cut 10000 tonnes |
| Duffy et al ^73^(2023), Canada | Pulse oximeters  (not mentioned) | LCA (“methods accepted by international standards and guidance") | One day of pulse oximetry measurement in emergency department | 23·4 kg CO₂e for one day of pulse oximeter use | 3·9 kg CO₂e per use (low use)  4·9 kg CO₂e per use (moderate use)  5·7 kg CO₂e per use (high use) | Production (74%) | Cleaning phase (99%) | Reusable pulse oximeters have 2-5-fold lower carbon footprint than single-use, producing fewer emissions after only 2.3 uses per day |
| Griffing and Overcash^31^ (2023), USA | Incontinence underpads  (not mentioned) | LCA (ISO 14040/ 14044) | 1000 reuses of incontinence underpads in hospital and long-term care facilities | 880 kg CO₂e from 2120 pad uses, equivalent to 1000 reusable uses | 340 kg CO_2_e per 1000 uses | Not mentioned | Laundry | Reusable incontinence underpads have 61% lower carbon footprint |
| Hemberg et al^72^ (2023), Sweden | Central Venous Catheter/ CVC insertion kits  (Scissors: Mayo. Needle holder: Mayo-Hegar) | LCA (IMPACT 2002+) | One CVC insertion | 2·3 kgCO_2_e | 0·24 kgCO_2_e | Production of sterile, single-use textiles (65-85%) | Washer-disinfector energy  Autoclave energy | Use of CVC-insertion kits containing reusable metal instruments and reusable textiles reduce carbon footprints in European setting |
| Kemble et al^32^ (2023), USA | Flexible cystoscopes  (Single-use: Ambu aScope. Reusable: Olympus CYF-V2) | LCA (not mentioned) | One case | 2·40 kg CO₂e per case | 0·53 kg CO₂e per case | Manufacturing | Energy consumption of reprocessing  Manufacturing  Repair and repackaging | Carbon footprint of reusable flexible cystoscopes is lower than single-use cystoscopes over the lifecycle of the devices, regardless of case volume |
| Lichtnegger^74^ et al (2023), Austria | Intermittent pneumatic compression (IPC) sleeves  (Single-use: Kendall SCD sleeves type 9529. Reusable: type 9529R) | LCA (ISO 14044) | Treatment of 5 patients with IPC | 7 kg CO₂e for treatment of 5 patients with IPC | 4·2 kg CO₂e for treatment of 5 patients with IPC | Manufacturing | Reprocessing | Reprocessed IPC sleeves have 40% lower carbon footprint compared to single-use IPC sleeves |
| Luo et al^67^ (2023), China | Various face masks  (not mentioned) | LCA (ISO 14067) | Masks used by 100 individuals over a period of one month; reusable masks discarded after 6 days of use and 5 washes | 128·926 kg CO₂e for masks used by 100 individuals over one month (low risk scenario)  0·043 kg CO₂e per medical mask (low risk scenario)  154·328 kg CO₂e for surgical masks used by 100 individuals over one month (medium risk scenario)  641·249 kg CO₂e for KN95 used by 100 individuals over one month (medium risk scenario) | 285·484 kg CO₂e for reusable cotton masks used by 100 individuals over one month (low risk scenario)  0·488 kg CO₂e per mask | Raw material processing Mask production | Cleaning  Production and supply processes of water and detergent  Laundry wastewater treatment | Carbon footprint of single-use medical masks is lower than that of cotton masks due to cotton cultivation stage |
| Meissner et al^62^ (2023), Austria | Surgical staplers  (Single-use: Echelon Flex stapler with Echelo Endopath Staple Line Reinforcement. Reusable: multi-use Signia stapler with Tri-Staple reinforced reloads with buttressing incorporated) | Product Material Analysis; greenhouse gas emission analysis limited to impact of lithium in power supply of staplers | All staple firings per surgical procedure (sleeve gastrectomy;  Roux-en-Y gastric bypass +/- buttressing) | 5·904 g CO₂e from lithium content in stapler’s power supply to perform all staple firings for one surgical procedure | 0·018 g CO₂e from lithium content in stapler’s power supply to perform all staple firings for one surgical procedure | Four cells of lithium content | Two cells of lithium content | Carbon footprint per surgical procedure based on lithium content of stapler’s power supply  Reusable staplers have less lithium  Reuse of rechargeable lithium-ion batteries should be prioritised over recycling of primary batteries |
| Quintana-Gallardo et al^60^ (2023), Spain | Surgical gowns  (not mentioned) | LCA (ISO 14040/ ISO 14025) | One surgical gown; various types assessed | 0·14-0·57 kg CO₂e per gown type | Not mentioned | Raw materials Energy  Transportation | Not mentioned | More sophisticated gowns such as reinforced sterile surgical gowns, had higher carbon footprint  Non-sterile gowns had the lowest individual footprint, but contributed most to impact from hospital in study due to high annual consumption |
| Rouviere et al^52^ (2023), France | Laryngoscope blades  (Single-use: "usual supplier to our healthcare facility". Reusable Blades: ProAct (Smoothline), Teleflex (Maxlite), Santelec (Sirius XL), Heine (Classic+)) | LCA (not mentioned) | Single intubation | Emissions not provided | Emissions not provided | Manufacturing optic fibre (64%)  Manufacturing stainless steel (25%)  Waste (11%) | Packaging (34%)  Washing (32%)  Sterilisation (31%) | Switching to reusable saves 1·54 kgCO2e per intubation |
| Snigdha et al^66^ (2023), India | Personal Protective Equipment body coveralls  (“market-representative”) | LCA (ISO 14040/ 14044) | 1000kg for each type of body overall | 4673·7 kg CO₂e for disposable PPE product life | 714·2 kg CO₂e for reusable PPE product life | Not mentioned | Not mentioned | Reusable PPE body coverall has lower carbon footprint |
| Chang et al^33^ (2024), USA | Operating room bed covers and lift sheets  (not mentioned) | LCA (ISO 14040/ 14044) | 50 covered operating beds | 64·99 kg CO₂e per covered operating bed  108·98 kg CO₂e per covered operating bed plus incineration | 19·83 kg CO₂e per covered operating bed | Raw material extraction (59·85 kg CO₂e)  Incineration (46·52 kg CO₂e) Transportation (2·61 kg CO₂e for 50 lift single-use) | Laundering 50 times (12·12 kg CO₂e)  Raw material extraction (6·71 kg CO₂e)  Landfill disposal (0·69 kg CO₂e) | Reusable fabric-based OR bed cover and lift sheet laundered 50 times has lower carbon footprint than single-use  Disposal of plastic-based single-use OR bed covers and life sheets as biohazard waste for incineration increases carbon footprint six-fold |
| Chen et al^43^ (2024), Australia | Sterile light handles  (not mentioned) | LCA (not mentioned) | 12 months of sterile light handle use for two hospitals | 3241·2 kg CO₂e for 12 months of sterile light handle use in Hospital A  187·3 kg CO₂e for 12 months of sterile light handle use in Hospital B | 3399·5 kg CO₂e for 12 months of sterile light handle use in Hospital A  1143·9 kg CO₂e for 12 months of sterile light handle use in Hospital A | Not mentioned | Energy requirements for re-sterilisation | Carbon footprint of reusable equipment is higher because of energy use for re-sterilisation and the weight of reusable devices  Sterilising reusable light handles as part of hollow ware set rather than as individual items, and increasing renewable energy mix, would decrease carbon footprint |
| Donahue et al^34^ (2024), USA | Surgical caps  (not mentioned) | Process-based LCA (ISO 14044) | Coverage of one surgical team (surgeon, circulator, scrub nurse, anaesthesiologist), for one year, completing 417 procedures per year | 49·039 kg CO₂e for coverage for surgical team for one year | 10·176 kg CO₂e for coverage for surgical team for one year | Not mentioned | Not mentioned | Disposable bouffant caps have 79% higher carbon footprints than reusable cotton caps in most common use scenarios |
| Hansell et al^44^ (2024), Australia | Intermittent pneumatic compression/ IPC sleeves  (not mentioned) | LCA (not mentioned) | Single pair of IPC devices, used once continuously on a single patient before disposal | 432·2 g CO₂e for IPC use for one patient | Not mentioned | Not mentioned | Not mentioned | Reducing unnecessary IPC use in the ICU setting can result in 51·8 kg CO₂e annual saving |
| Kidane et al^35^ (2024), USA | Flexible laryngoscopes  (Single-use: aScope 4 RhinoLaryngo Sim. Reusable: Olympus ENF-V2) | LCA (ISO 14040) | 1308 flexible laryngoscopy examinations | 2619 kg CO₂e for 1308 examinations | 1816 kg CO_2_e for 1308 examinations | Manufacturing and production (79%) Incineration emits more than waste to landfill | PPE production and disposal used during reprocessing (63%) | Reusable laryngoscope has lower carbon footprint compared to single-use |
| Lehane et al^36^ (2024), USA | Paediatric surgical kit  (not mentioned) | Greenhouse Gases, Regulated Emissions, and Energy use in Transportation (GREET) | Use of paediatric surgical kit, scaled to annual surgical volumes at a single freestanding children's hospital | 3946·4 g CO₂e for one surgical kit  86 820 800 g CO₂e for one year of kits (n=22 000) | Not mentioned | Surgical gowns  Central supply room wraps  Drapes | Not mentioned | Reducing the number of gowns contained in surgical kits decreases carbon footprint by 11%. Assessment of surgical kit content, using sustainable alternatives, reducing quantities and omitting unnecessary products, can lower carbon footprint |
| Lightfoot et al^42^ (2024), Australia | Anaesthetic drug trays  (Multigate) | LCA (ISO 14040/ 14044) | One single-use disposable drug tray; 10 types compared | 33–454 g CO₂e per tray depending on tray | Not mentioned | Not mentioned | Not mentioned | Mass of tray predictor of carbon emissions for landfill, recycling and incinerations, regardless of material makeup  Manufacturers should develop the lightest trays possible  Anaesthetists should reduce the number of trays used |
| Lopez-Munoz et al^65^ (2024), Spain | Duodenoscope for Endoscopic Retrograde Cholangiopancreatography/ ERCP  (not mentioned) | LCA (ISO 14040) | Five ERCPs per week and reusable duodenoscope lifetime of 8 years totalling 1600 procedures | 7·9 kg CO₂e per ERCP (Brand A)  6·6 kg CO₂e per ERCP (Brand B)  12640 kg CO₂e for 1600 ERCPs (Brand A)  10512 kg CO₂e for 1600 ERCPs (Brand B) | 0·1 kg CO₂e per use reusable; 152 kg CO₂e over lifetime | Manufacturing (3·7 kg CO₂e, Brand A) Manufacturing (3·2 kg CO₂e, Brand B) | Manufacturing (22·8 kg CO₂e)  Reprocessing and disinfection (84%) | Widespread adoption of single-use duodenoscopes would result in increased carbon footprint |
| Massart et al^64^ (2024), France | Bronchoscope  (Single-use: Bronchoflex agile, TSC. Reusable: Pentax FI-16RBS) | LCA/Pragmatic, eco-audit study (not mentioned) | Whole bronchoscopy activity in ICU during 2022 totalling 110 bronchoscopies) | 3·82 kg CO_2_e per bronchoscopy  418 kg CO_2_e for 110 procedures performed with single-use bronchoscope | 3·09 kg CO₂e per bronchoscopy  185·5 kg CO₂e for 60 bronchoscopies | Camera (33%) Printed board (7%) | Disinfection (>95%) | Reusable bronchoscopes have lower carbon footprint when used more than 50 times a year |
| Pioche et al^54^ (2024), France | Gastroscopes  (Single-use: Ambu aScope Gastro. Reusable: Olympus H190) | LCA (ISO 14040/ ISO 9001/ 14001) | Provision of one upper gastrointestinal endoscopy | 10·9 kg CO₂e per endoscopy | 0·018 kg CO₂e per endoscopy  4·7 kg CO₂e per endoscopy with decontamination included | Production (56%) | Reprocessing (45%) | Single-use endoscope carries a 2·5-fold greater carbon footprint per procedure.  Unnecessary examination must be avoided to reduce overall carbon footprint |
| Rizan^50^ (2024), UK | Robotic hybrid 5mm ports (Surgical Innovations Ltd.) | LCA (ISO 14044) | One use of hybrid 5mm robotic port, compared with individually wrapped single-use 5mm port | 816 g CO_2_e per use of port | 143 g CO_2_e per use of port | Not mentioned | Not mentioned | Switching from single-use to hybrid equivalent ports was found to save 2·46 kg CO_2_e per operation and 3050 Mt CO_2_e if applied to all global robotic procedures annually |
| Thöne et al^59^ (2024), Germany | Flexible ureteroscope  (not mentioned) | LCA (ISO 14040/ 14044) | Use of flexible ureteroscope for one hour | 4·93 kg CO₂e per use | 1·24 kg CO₂e per use | Production (3·5 kg CO₂e) Disposal (1·2 kg CO₂e) Use (0·14 kg CO₂e) | Reprocessing (0·88 kg CO₂e) Production (0·16 kg CO₂e)  Maintenance (0·083 kg CO₂e) | Eight uses of reusable ureteroscope suffices for reusable devices to have a lower impact than single-use |
| BP (blood pressure); CO₂ (carbon dioxide); CO₂e (carbon dioxide equivalent); CT (computed tomography); CVC (central venous catheter); ERCP (endoscopic retrograde cholangiopancreatography); FFP2 (filtering facepiece, class 2); g (gram); GWP (global warming potential); ICU (intensive care unit); ISO (International Organization for Standardization); kg (kilogram); LCA (Life Cycle Assessment); LMA (laryngeal mask airway); MRI (magnetic resonance imaging); Mt (Metric tonne); N₂O (nitrous oxide); NHS (National Health Service); NOx (nitrogen oxides); PAS (Product Attribute Scoring); PEF (Product Environmental Footprint); PVC (polyvinyl chloride); US (United States). | | | | | | | | |

**References**

1. Kümmerer K, Dettenkofer M, Scherrer M. Comparison of reusable and disposable laparatomy pads. The International Journal of Life Cycle Assessment. 1996;1(2):67-73.

2. Ison E, Miller A. The use of LCA to introduce life-cycle thinking into decision making for the purchase of medical devices in the NHS. Journal of Environmental Assessment Policy and Management. 2000;2(4):453-76.

3. McGain F, McAlister S, McGavin A, Story D. The financial and environmental costs of reusable and single-use plastic anaesthetic drug trays. Anaesth Intensive Care. 2010;38(3):538-44.

4. Eckelman M, Mosher M, Gonzalez A, Sherman J. Comparative life cycle assessment of disposable and reusable laryngeal mask airways. Anesth Analg. 2012;114(5):1067-72.

5. Grimmond T, Reiner S. Impact on carbon footprint: a life cycle assessment of disposable versus reusable sharps containers in a large US hospital. Waste Manag Res. 2012;30(6):639-42.

6. McGain F, McAlister S, McGavin A, Story D. A life cycle assessment of reusable and single-use central venous catheter insertion kits. Anesth Analg. 2012;114(5):1073-80.

7. Ibbotson S, Dettmer T, Kara S, Herrmann C. Eco-efficiency of disposable and reusable surgical instruments—a scissors case. The International Journal of Life Cycle Assessment. 2013;18(5):1137-48.

8. Sørensen BL, Wenzel H. Life cycle assessment of alternative bedpans – a case of comparing disposable and reusable devices. Journal of Cleaner Production. 2014;83:70-9.

9. Pourzahedi L, Eckelman MJ. Environmental life cycle assessment of nanosilver-enabled bandages. Environmental Science & Technology.49(1):361-8.

10. Campion N, Thiel CL, DeBlois J, Woods NC, Landis AE, Bilec MM. Life cycle assessment perspectives on delivering an infant in the US. The Science of the total environment. 2012;425:191-8.

11. Esmaeili A, Twomey JM, Overcash MR, Soltani SA, McGuire C, Ali K. Scope for energy improvement for hospital imaging services in the USA. J Health Serv Res Policy. 2015;20(2):67-73.

12. McGain F, Story D, Lim T, McAlister S. Financial and environmental costs of reusable and single-use anaesthetic equipment. Br J Anaesth. 2017;118(6):862-9.

13. Unger SR, Hottle TA, Hobbs SR, Thiel CL, Campion N, Bilec MM, et al. Do single-use medical devices containing biopolymers reduce the environmental impacts of surgical procedures compared with their plastic equivalents? Journal of Health Services Research and Policy. 2017;22(4):218-25.

14. Davis NF, McGrath S, Quinlan M, Jack G, Lawrentschuk N, Bolton DM. Carbon Footprint in Flexible Ureteroscopy: A Comparative Study on the Environmental Impact of Reusable and Single-Use Ureteroscopes. J Endourol. 2018;32(3):214-7.

15. Martin M, Mohnke A, Lewis GM, Dunnick NR, Keoleian G, Maturen KE. Environmental Impacts of Abdominal Imaging: A Pilot Investigation. J Am Coll Radiol. 2018;15(10):1385-93.

16. Sherman JD, Raibley LAt, Eckelman MJ. Life Cycle Assessment and Costing Methods for Device Procurement: Comparing Reusable and Single-Use Disposable Laryngoscopes. Anesth Analg. 2018;127(2):434-43.

17. Vozzola E, Overcash M, Griffing E. Environmental considerations in the selection of isolation gowns: A life cycle assessment of reusable and disposable alternatives. Am J Infect Control. 2018;46(8):881-6.

18. McPherson B, Sharip M, Grimmond T. The impact on life cycle carbon footprint of converting from disposable to reusable sharps containers in a large US hospital geographically distant from manufacturing and processing facilities. PeerJ. 2019;7:e6204-e.

19. Willskytt S, Tillman A-M. Resource efficiency of consumables – Life cycle assessment of incontinence products. Resources, Conservation and Recycling. 2019;144:13-23.

20. Donahue LM, Hilton S, Bell SG, Williams BC, Keoleian GA. A comparative carbon footprint analysis of disposable and reusable vaginal specula. Am J Obstet Gynecol. 2020;223(2):225.e1-.e7.

21. Leiden A, Cerdas F, Noriega D, Beyerlein J, Herrmann C. Life cycle assessment of a disposable and a reusable surgery instrument set for spinal fusion surgeries. Resources, Conservation and Recycling. 2020;156:104704.

22. Sanchez SA, Eckelman MJ, Sherman JD. Environmental and economic comparison of reusable and disposable blood pressure cuffs in multiple clinical settings. Resources, Conservation and Recycling. 2020;155:104643.

23. Vozzola E, Overcash M, Griffing E. An Environmental Analysis of Reusable and Disposable Surgical Gowns. AORN Journal. 2020;111(3):315-25.

24. Baxter NB, Yoon AP, Chung KC. Variability in the Use of Disposable Surgical Supplies: A Surgeon Survey and Life Cycle Analysis. J Hand Surg Am. 2021.

25. Grimmond TR, Bright A, Cadman J, Dixon J, Ludditt S, Robinson C, et al. Before/after intervention study to determine impact on life-cycle carbon footprint of converting from single-use to reusable sharps containers in 40 UK NHS trusts. BMJ open. 2021;11(9):e046200-e.

26. Jamal H, Lyne A, Ashley P, Duane B. Non-sterile examination gloves and sterile surgical gloves: which are more sustainable? J Hosp Infect. 2021;118:87-95.

27. van Straten B, Ligtelijn S, Droog L, Putman E, Dankelman J, Weiland NHS, et al. A life cycle assessment of reprocessing face masks during the Covid-19 pandemic. Sci Rep. 2021;11(1):17680.

28. Atilgan Turkmen B. Life cycle environmental impacts of disposable medical masks. Environmental Science & Pollution Research.29(17):25496-506.

29. Boberg L, Singh J, Montgomery A, Bentzer P. Environmental impact of single-use, reusable, and mixed trocar systems used for laparoscopic cholecystectomies. PLoS ONE [Electronic Resource].17(7):e0271601.

30. Burguburu A, Tanné C, Bosc K, Laplaud J, Roth M, Czyrnek-Delêtre M. Comparative life cycle assessment of reusable and disposable scrub suits used in hospital operating rooms. Cleaner Environmental Systems. 2022;4:100068.

31. Le NNT, Hernandez LV, Vakil N, Guda N, Patnode C, Jolliet O. Environmental and health outcomes of single-use versus reusable duodenoscopes. Gastrointestinal Endoscopy. 2022;17:17.

32. Maloney B, McKerlie T, Nasir M, Murphy C, Moi M, Mudalige P, et al. The environmental footprint of single-use versus reusable cloths for clinical surface decontamination: a life cycle approach. Journal of Hospital Infection.130:7-19.

33. Rizan C, Bhutta MF. Environmental impact and life cycle financial cost of hybrid (reusable/single-use) instruments versus single-use equivalents in laparoscopic cholecystectomy. Surgical Endoscopy.36(6):4067-78.

34. Agarwal D, Bharani T, Armand W, Slutzman JE, Mullen JT. Reusable scrub caps are cost-effective and help reduce the climate footprint of surgery. Langenbeck's Archives of Surgery. 2023;408(1):358.

35. Baboudjian M, Pradere B, Martin N, Gondran-Tellier B, Angerri O, Boucheron T, et al. Life Cycle Assessment of Reusable and Disposable Cystoscopes: A Path to Greener Urological Procedures. European Urology Focus. 2023;9(4):681-7.

36. Byrne C, Pley C, Schorscher P, Brandon Z, Gatumbu P, Mallinson C, et al. A mixed-methods analysis of the climate impact, acceptability, feasibility and cost of switching from single-use pulp to reusable plastic trays in a large NHS trust. Future Healthc J. 2023;10(2):157-60.

37. Cohen ES, Djufri S, Bons S, Knoppert MR, Hehenkamp WJK, Kouwenberg LHJA, et al. Environmental Impact Assessment of Reusable and Disposable Surgical Head Covers. JAMA Surgery. 2023;158(11):1216-7.

38. Duffy JS, J. Thiel, CL. Landes, M. Sustainable purchasing practices: A comparison of single-use and reusable pulse oximeteres in the emergency department. Western Journal of Emergency Medicine: Integrating Emergency Care with Population Health. 2023;24.

39. Griffing E, Overcash M. Reusable and Disposable Incontinence Underpads: Environmental Footprints as a Route for Decision Making to Decarbonize Health Care. J Nurs Care Qual. 2023;38(3).

40. Hemberg L, Wessberg N, Leire C, Bentzer P. Environmental impact of single-use and reusable items in central venous catheter insertion kits: a life cycle assessment. Intensive Care Med. 2023;49(6):662-4.

41. Kemble JP, Winoker JS, Patel SH, Su ZT, Matlaga BR, Potretzke AM, et al. Environmental impact of single-use and reusable flexible cystoscopes. BJU Int. 2023;131(5):617-22.

42. Lichtnegger S MM, Paolini F, Veloz A, Saunders R. Comparative Life Cycle Assessment Between Single-Use and Reprocessed IPC Sleeves. Risk Management Healthcare Policy. 2023;16.

43. Luo Y, Yu M, Wu X, Ding X, Wang L. Carbon footprint assessment of face masks in the context of the COVID-19 pandemic: Based on different protective performance and applicable scenarios. Journal of Cleaner Production. 2023;387:135854.

44. Meissner M HJ, Silas U, Saunders R. Evaluating the Environmental Impact of Single-Use and Multi-Use Surgical Staplers with Staple Line Buttressing in Laparoscopic Bariatric Surgery. Risk Management Healthcare Policy. 2023;16.

45. Quintana-Gallardo A, del Rey R, González-Conca S, Guillén-Guillamón I. The Environmental Impacts of Disposable Nonwoven Fabrics during the COVID-19 Pandemic: Case Study on the Francesc de Borja Hospital. Polymers. 2023;15(5):1130.

46. Rouvière N, Chkair S, Auger F, Cuvillon P, Leguelinel-Blache G, Chasseigne V. Reusable laryngoscope blades: a more eco-responsible and cost-effective alternative. Anaesthesia Critical Care & Pain Medicine. 2023;42(5):101276.

47. Snigdha, Hiloidhari M, Bandyopadhyay S. Environmental footprints of disposable and reusable personal protective equipment ‒ a product life cycle approach for body coveralls. Journal of Cleaner Production. 2023;394:136166.

48. Chang JH, Woo KP, Silva de Souza Lima Cano N, Bilec MM, Camhi M, Melnyk AI, et al. Does reusable mean green? Comparison of the environmental impact of reusable operating room bed covers and lift sheets versus single-use. The Surgeon. 2024;22(4):236-41.

49. Chen S, McAlister S, Colagiuri P, Pickles K, Barratt AL. Switching to reusable operating theatre equipment: lessons learnt from sterile light handle projects in two Australian hospitals. ANZ Journal of Surgery. 2024;95(1-2):216-21.

50. Donahue LM, Petit HJ, Thiel CL, Sullivan GA, Gulack BC, Shah AN. A Life Cycle Assessment of Reusable and Disposable Surgical Caps. J Surg Res. 2024;299:112-9.

51. Hansell L, Delaney A, Milross M, Henderson E. Reducing unnecessary use of intermittent pneumatic compression in intensive care: A before-and-after pilot study with environmental perspective. Aust Crit Care. 2025;38(2).

52. Kidane J, Thiel CL, Wang K, Rosen CA, Gandhi S. A Comparison of Environmental Impacts Between Reusable and Disposable Flexible Laryngoscopes. The Laryngoscope. 2025;135(5):1666-73.

53. Lehane A, Sullivan GA, Dunn J, Perez M, Smith CJ, Raval MV. Life Cycle Analysis of a Pediatric Surgical Kit—A Target to Reduce Operating Room Waste. JAMA Surgery. 2024;159(12):1436-8.

54. Lightfoot SJ, Grant T, Boyden A, McAlister S. Single-use synthetic plastic and natural fibre anaesthetic drug trays: a comparative life cycle assessment of environmental impacts. British Journal of Anaesthesia. 2024;133(6):1465-77.

55. López-Muñoz P, Martín-Cabezuelo R, Lorenzo-Zúñiga V, García-Castellanos M, Vilariño-Feltrer G, Tort-Ausina I, et al. Environmental footprint and material composition comparison of single-use and reusable duodenoscopes. Endoscopy. 2024;57(02):116-23.

56. Massart N, Millet C, Beloeil H, Fillatre P, Rouxel C, Daudin M, et al. How green is my reusable bronchoscope? Anaesthesia Critical Care & Pain Medicine. 2024;43(5):101420.

57. Pioche M, Pohl H, Cunha Neves JA, Laporte A, Mochet M, Rivory J, et al. Environmental impact of single-use versus reusable gastroscopes. Gut. 2024;73(11):1816-22.

58. Rizan C. Environmental impact of hybrid (reusable/single-use) ports versus single-use equivalents in robotic surgery. J Robot Surg. 2024;18(1):155.

59. Thöne M, Lask J, Hennenlotter J, Saar M, Tsaur I, Stenzl A, et al. Potential impacts to human health from climate change: A comparative life-cycle assessment of single-use versus reusable devices flexible ureteroscopes. Urolithiasis. 2024;52(1):166.
