# Supplementary material for: The carbon footprints of single-use and reusable medical devices: a systematic review
Source: BMJ Open. 2025 Dec 19;15(12):e108446. doi: 10.1136/bmjopen-2025-108446 (PMC12716512; doi:10.1136/bmjopen-2025-108446)
Supplement: Supplementary file 2 [file bmjopen-2025-108446.reviewer_comments.pdf]

PEER REVIEW HISTORY

BMJ Open publishes all reviews undertaken for accepted manuscripts. Reviewers are asked to complete a checklist review form and are provided with free text boxes to elaborate on their assessment. These free text comments are reproduced below.

ARTICLE DETAILS

Title (Provisional)

The carbon footprints of single-use and reusable medical devices: A systematic review

Authors

Booth, Amy; Chowaniec, Monika; Goyal, Sanay; Faulkner, Stuart; Shaw, Sara

---

VERSION 1 - REVIEW

**Reviewer**	**1**
**Name**	**Richie, Cristina**
**Affiliation**	**university of edinburgh**
**Date**	**26-Aug-2025**
**COI**	**None**

This research makes an important contribution to the existing literature. It is well researched, detailed, clear to read and has more than sufficient documentation. The authors have presented the information with significant normative implications for sustainable health care.

**Reviewer**	**2**
**Name**	**LaRoche, Jason**
**Affiliation**	**Janssen Research and Development LLC, Janssen Clinical Innovation**
**Date**	**29-Oct-2025**
**COI**	**None**

Dear Dr. Amy Booth,

Thank you for submitting your manuscript for review. I feel that your team's research makes a very valuable contribution to the scope of knowledge around medical device circularity. Being a co-founder and work package lead for the Horizon's Europe funded Digital Health in

the Circular Economy (DiCE) project, I wish we had such a systematic review of the current literature available to us at the start, as we've had to build this knowledge incrementally.

I have enclosed my comments in the pdf copy of the manuscript. Overall, I found your manuscript to be very well written and only found one section where some clarification is needed. I have also suggested a modified definition for a reprocessed/reused device that better aligns with a standard definition developed by the DiCE project to address a lack of harmonized terminology we see across various EU policies and recommendations that touch on medical device manufacture, use, reprocessing, and disposition.

The majority of my comments reflect questions that arise in my mind as I read you results, and generally present opportunities for you to enhance the value of the insights you provide. For example, you provide a breakdown on the data sources relied upon by the different research publications that fell within your scope. You had one such grouping, "national or regional databases", and it would be interesting to understand if the European Platform on LCA's International Life Cycle Data System is part of that sub-set. The European Commission established that data set to serve as a "single-source of truth" in guiding EU policy making, so it would be interesting to understand the uptake of that data source by the broader ecosystem. Please accept such commentary as suggestions to enhance the value of the research, but I respect that data limitations and/or the need to maintain a concise focus for the manuscript may prevent exploration of tangential opportunities.

Kind Regards,

Jason LaRoche

Jason LaRoche

Director, Innovative Health – Environmental Sustainability

Business Growth, Innovation and Advocacy (BGIA)

J&J Innovative Medicine

**** **The reviewer provided a marked copy with additional comments. Please contact the publisher for full details.** ****

VERSION 1 - AUTHOR RESPONSE

Reviewer 1: Dr Cristina Richie (University of Edinburgh)

Comment:

This research makes an important contribution to the existing literature. It is well researched, detailed, clear to read and has more than sufficient documentation. The authors have presented the information with significant normative implications for sustainable health care.

Response:

We sincerely thank Dr Richie for her positive and encouraging comments. No revisions were requested in this section.

Reviewer 2: Dr Jason LaRoche (Janssen R&D; Delft University of Technology)

Thank you for submitting your manuscript for review. I feel that your team's research makes a very valuable contribution to the scope of knowledge around medical device circularity. Being a co-founder and work package lead for the Horizon's Europe funded Digital Health in the Circular Economy (DiCE) project, I wish we had such a systematic review of the current literature available to us at the start, as we've had to build this knowledge incrementally.

I have enclosed my comments in the pdf copy of the manuscript. Overall, I found your manuscript to be very well written and only found one section where some clarification is needed. I have also suggested a modified definition for a reprocessed/reused device that better aligns with a standard definition developed by the DiCE project to address a lack of harmonized terminology we see across various EU policies and recommendations that touch on medical device manufacture, use, reprocessing, and disposition.

The majority of my comments reflect questions that arise in my mind as I read you results, and generally present opportunities for you to enhance the value of the insights you provide. For example, you provide a breakdown on the data sources relied upon by the different research publications that fell within your scope. You had one such grouping, "national or regional databases", and it would be interesting to understand if the European Platform on LCA's International Life Cycle Data System is part of that sub-set. The European Commission established that data set to serve as a "single-source of truth" in guiding EU policy making, so it would be interesting to understand the uptake of that data source by the broader ecosystem. Please accept such commentary as suggestions to enhance the value of the research, but I respect that data limitations and/or the need to maintain a concise focus for the manuscript may prevent exploration of tangential opportunities.

Response:

We thank Dr LaRoche for his thoughtful and detailed feedback, which has allowed us to strengthen our manuscript and value of this research. We detail our responses to each point below.

Comment 1:

"Reusable / reprocessed device: A device that is cleaned, sterilised, or otherwise treated for safe reuse across multiple patients or procedures."

Suggest aligning with harmonized terminology being developed by the EU-funded Digital Health in the Circular Economy project which also includes mention of testing and restoring the technical and functional safety of the device. Our intent is to use this harmonized terminology to support broader policy recommendations and address inconsistencies in terminology across different EU regulations. DiCE term: A process carried out on a used device in order to allow its safe reuse including cleaning, disinfection, sterilisation and related procedures, as well as testing and restoring the technical and functional safety of the used device.

Response:

We appreciate this suggestion and have revised the definition to better align with the emerging harmonised terminology proposed by the DiCE project. The revised text now reads:

"**Reusable / reprocessed device:** A device that undergoes processes after use to ensure its safe reuse across multiple patients or procedures, including cleaning, disinfection, sterilisation and related procedures as well as testing and restoring the technical and functional safety of the used device." (Page 5, Box 1).

Comment 2:

"national or regional databases"

Were you able to specifically breakout use of the European Platform on LCA's International Life Cycle Data System? The European commission established the dataset to serve as a single-source of truth in EU policy shaping. It would be interesting to understand how well leveraged this dataset is today compared with competing data sources.

Response:

Thank you for this insightful question. Only one of the included studies (Burguburu et. al.) explicitly cited the ILCD as a data source. We have added this database as an example in the Results section:

"Inventory data sources (e.g., for emission factors) varied, with many studies using EcoInvent (n=38); national or regional databases (n=20) including the US Life Cycle Inventory, the UKs Department for Environment, Food and Rural Affair, the EU's International Life Cycle Data system, and China's Life Cycle Database; and industry datasets (n=10)." (Page 11, Line 241).

Comment 3:

"Use" can be a broad term that could involve the environmental impacts of the device's use alone (footprint) but also include a device's influence (handprint) on the larger care pathway. It would be useful to define the term "use" as you apply it here. It would be interesting to further breakout footprint vs. handprint studies to understand the growth of more holistic approaches. For example, in the EU-funded Digital Health in the Circular Economy project, it was found that technical challenges prevent many more advanced surgical devices from being sufficiently cleaned and sterilized for re-use. The footprint of these devices is substantial which might lead one to believe we should reduce use of these devices. However, a more holistic study found that use of these devices significantly reduced hospitalization times and risk of post-surgery infections compared with other established options. The handprint of these devices more than offset their footprint, which indicates that their use should be encouraged rather than reduced. With France implementing product carbon footprint requirements in all of their public tenders in 2026 (the NHS will not be far behind with their 2028 target), the reliance on PCFs which focus only on the footprint pose the risk of disadvantaging products that actually do a better job reducing the overall footprint of healthcare.

Response:

Thank you for highlighting this important distinction. In our analysis, the term 'use', reflecting how the bulk of studies employed the term, refers specifically to the direct carbon footprint associated

with the operation or clinical application of the device, such as energy consumption during use, corresponding to the ‘footprint’ dimension. It does not encompass the wider care pathway benefits or trade-offs (i.e., the handprint) such as improved patient outcomes, reduced length of stay, or avoided downstream resource use.

We have clarified what ‘use’ encompasses in the Results section:

“use, **referring specifically to the carbon footprint associated with the operation or clinical application of the device…”** (Page 13, Line 252)

Additionally, to acknowledge this broader perspective, we have added the following statement in the Discussion section:

“Finally, while our review primarily assessed the carbon footprint associated with medical devices, there is a need for future work to integrate a ‘handprint’ approach, that is, examining the procedural performance of the device, and how device use influences patient outcomes and may reduce the need for further, more carbon-intensive care if the device were not used. Such analysis would capture more holistic climate and clinical co-benefits and trade-offs. For example, the findings reported by Leiden et al., where single-use surgical equipment shortened operating times and reduced theatre energy use, illustrate how procedural efficiency can generate positive handprint effects that offset some of the carbon footprint of devices.” (Page 32, Lines 395-401).

Comment 4:

“Reprocessing of reusable”

In the earlier metrics, you reported 27 in-scope papers from the EU and 23 from the US. On the topic of reprocessing, did you see a substantial difference in source (EU vs US)? The reason I ask is that the US is a large market regulated by the US FDA alone. This has allowed reprocessing to proliferate across the US with reprocessors like Striker and Sterilmed widely collecting, reprocessing, and selling reprocessed devices back to hospitals and clinics. Meanwhile, in Europe a very fragmented regulatory ecosystem has served as a major point of friction preventing the capacity for reprocessing to sufficiently scale. I'm curious if the lack of reprocessing across Europe is reflected in a reduced number of papers on the topic from the EU region compared with the US where reprocessing data is more readily available.

Response:

This is a helpful observation. In our review, we examined whether reprocessing was included within the carbon modelling system boundaries of each study, rather than how or by whom reprocessing was undertaken. In our study, reprocessing was defined as the cleaning, sterilisation, or decontamination of devices for safe reuse, typically undertaken on-site (e.g., autoclave reprocessing) or occasionally by third-party providers.

Across both EU and US studies, where ‘reprocessing’ was included in carbon modelling boundaries, it generally referred to on-site or small-scale reprocessing rather than large-scale commercial reprocessing systems. Consequently, our data did not permit meaningful comparison of reprocessing prevalence and market dynamics between the EU and US contexts.

We have clarified what reprocessing encompasses in the Results section:

“reprocessing of reusable devices, referring to the cleaning, disinfection, and sterilisation of devices for safe reuse, typically on-site or by third-party contractors” (Page 13, Line 254-5).

To acknowledge this point, we have also added the following statement in the Discussion section:

“For example, although more than half of the studies incorporated reprocessing within their carbon modelling boundaries, these typically referred to on-site or small-scale processes rather than large-scale commercial reprocessing. As such, regional differences in reprocessing regulatory environments, market dynamics, and large-scale reprocessing practices were not captured in this review and warrant future investigation.” (Page 31, Lines 385-389).

Comment 5 (Page 15, Line 284–285):

“Leiden et al showed single-use surgical equipment resulted in shorter operating times that lowered theatre energy use.”

This would be an example of a more holistic "handprint" study that I referenced in my earlier comments.

Response:

Thank you for this observation. We agree that the study by Leiden et al. provides an example of a more holistic ‘handprint’ perspective, where improved procedural efficiency and reduced operating time can offset some of the carbon footprint associated with single-use devices.

As noted in our response to Comment 3, we have included a discussion on this in the Discussion section:

“Finally, while our review primarily assessed the carbon footprint associated with medical devices, there is a need for future work to integrate a ‘handprint’ approach, that is, examining the procedural performance of the device, and how device use influences patient outcomes and may reduce the need for further, more carbon-intensive care if the device were not used. Such analysis would capture more holistic climate and clinical co-benefits and trade-offs. For example, the findings reported by Leiden et al., where single-use surgical equipment shortened operating times and reduced theatre energy use, illustrate how procedural efficiency can generate positive handprint effects that offset some of the carbon footprint of devices.” (Page 32, Lines 395-401).

Comment 6:

“For example, reprocessing contributed...”

I suggest adding clarification that "during the use phase, reprocessing contributed...". It will avoid a reader believing this included the manufacturing phase where reprocessing would appear to contribute more emissions than manufacturing itself. In a time when some bad actors cherry-pick statements that fit their agenda, it can avoid someone claiming that reprocessing is bad.

Response:

We agree and have revised the sentence for clarity:

“For example, reprocessing reusable devices for reuse during the use phase ...” (Page 14, Lines 307- 308).

Comment 7

"The European Union's Medical Device Regulation similarly allows reprocessing under defined conditions, although national implementation may vary."

I'm happy to see you call this out as a point of friction because EU MDR does indeed define circularity but leave operationalization to individual nation states. France, for example, has decided not to allow reprocessing removing a major EU market from consideration. Netherlands and Germany both allow reprocessing, but Netherlands imposes a far more stringent administrative burden. This prevents reprocessors from seeking economies of scale by having one EU process because that process would have to be geared around the most stringent administrative requirements of any one country, thus over-investing and wasting resources in less stringent markets.

Response:

We appreciate this opportunity to elaborate. We have expanded on this in the Discussion section to include the examples provided:

"The European Union's (EU's) Medical Device Regulation similarly allows reprocessing under defined conditions, although national implementation may vary.[84] For example, France currently prohibits reprocessing, whereas the Netherlands and Germany permit it, though the Netherlands imposes a more stringent administrative burden. Such regulatory divergence constrains economies of scale and limits the circularity potential of medical device reprocessing across EU markets." (Page 31, Lines 364-367).

VERSION 2 - REVIEW

**Reviewer**	**2**
**Name**	**LaRoche, Jason**
**Affiliation**	**Janssen Research and Development LLC, Janssen Clinical Innovation**
**Date**	**24-Nov-2025**
**COI**	

Dear Dr. Amy Booth,

Thank you for carefully considering and creatively addressing my prior feedback. I feel that the edits you have made in response to peer reviewers has enhanced what was already a very valuable paper. I have only two minor edits to recommend:

Page 17, Line 354: While France previously prohibited reprocessing, they recently announced that they are "in consultation" regarding reprocessing. The space is rapidly evolving so it can be difficult to keep up. We should probably rephrase this to say that "at

the time of authorship, France is now 'in consultation' to determine if they will allow reprocessing" or something similar. Given the timelines for legislative agendas and that this new status was only announced in recent weeks, it's probably safe to keep with this phrasing or something similar in your paper.

Page 18, Line 389: You make the statement, "For example, the findings reported by Leiden et al.,58 where single-use surgical equipment shortened operating times and reduced theatre energy use, illustrate how procedural efficiency can generate positive handprint effects that offset some of the carbon footprint of devices". I suggest updating this to read "offset some or all of the carbon footprint of devices" as there have been analyses that have shown offsets to exceed the environmental impact of both the manufacturing and use.

Thank you again for submitting this paper for review.

Kind Regards,

Jason LaRoche

Director, Innovative Health – Environmental Sustainability

Business Growth, Innovation and Advocacy (BGIA)

J&J Innovative Medicine

Global Development

**** **The reviewer provided a marked copy with additional comments. Please contact the publisher for full details.** ****

VERSION 2 - AUTHOR RESPONSE

Response to Reviewer 2

We thank Dr LaRoche for their additional feedback. Below we provide a point-by-point response and describe the corresponding revisions made to the manuscript.

Reviewer 2 comments

Thank you for carefully considering and creatively addressing my prior feedback. I feel that the edits you have made in response to peer reviewers has enhanced what was already a very valuable paper. I have only two minor edits to recommend:

Page 17, Line 354: While France previously prohibited reprocessing, they recently announced that they are "in consultation" regarding reprocessing. The space is rapidly evolving so it can be difficult to keep up. We should probably rephrase this to say that "at the time of authorship, France is now 'in consultation' to determine if they will allow reprocessing" or something similar. Given the timelines for legislative agendas and that this new status was only announced in recent weeks, it's probably safe to keep with this phrasing or something similar in your paper.

Page 18, Line 389: You make the statement, "For example, the findings reported by Leiden et al.,58 where single-use surgical equipment shortened operating times and reduced theatre energy use, illustrate how procedural efficiency can generate positive handprint effects that offset some of the carbon footprint of devices”. I suggest updating this to read “offset some or all of the carbon footprint of devices” as there have been analyses that have shown offsets to exceed the environmental impact of both the manufacturing and use.

Thank you again for submitting this paper for review.

Response

Thank you for your minor suggestions to improve clarity. We have adjusted the wording as recommended:

Page 17 Lines 354-356: For example, at the time of authorship, France is in consultation to determine if they will allow reprocessing; meanwhile, the Netherlands and Germany permit reprocessing, though the Netherlands imposes a more stringent administrative burden.

Page 18 Lines 390-391: For example, the findings reported by Leiden et al., where single-use surgical equipment shortened operating times and reduced theatre energy use, illustrate how procedural efficiency can generate positive handprint effects that offset some or all of the carbon footprint of devices.
